# Binding Affinity and Driving Forces for the Interaction of Calixarene-Based Micellar Aggregates With Model Antibiotics in Neutral Aqueous Solution

**DOI:** 10.3389/fchem.2020.626467

**Published:** 2021-01-14

**Authors:** Rossella Migliore, Giuseppe Granata, Andrea Rivoli, Grazia Maria Letizia Consoli, Carmelo Sgarlata

**Affiliations:** ^1^Dipartimento di Scienze Chimiche, Università Degli Studi di Catania, Catania, Italy; ^2^Istituto di Chimica Biomolecolare, Consiglio Nazionale delle Ricerche (CNR), Catania, Italy

**Keywords:** amphiphilic calixarenes, antibiotics, micelles, drug delivery systems, speciation, isothermal titration calorimetry, NMR, aqueous solution

## Abstract

The search for novel surfactants or drug delivery systems able to improve the performance of old-generation antibiotics is a topic of great interest. Self-assembling amphiphilic calix[4]arene derivatives provide well-defined nanostructured systems that exhibit promising features for antibiotics delivery. In this work, we investigated the capability of two micellar polycationic calix[4]arene derivatives to recognize and host ofloxacin, chloramphenicol, or tetracycline in neutral aqueous solution. The formation of the nanoaggregates and the host–guest equilibria were examined by nano-isothermal titration calorimetry, dynamic light scattering, and mono- and bi-dimensional NMR. The thermodynamic characterization revealed that the calix[4]arene-based micellar aggregates are able to effectively entrap the model antibiotics and enabled the determination of both the species and the driving forces for the molecular recognition process. Indeed, the formation of the chloramphenicol–micelle adduct was found to be enthalpy driven, whereas entropy drives the formation of the adducts with both ofloxacin and tetracycline. NMR spectra corroborated ITC data about the positioning of the antibiotics in the calixarene nanoaggregates.

## Introduction

The entrapment of target molecules in drug delivery systems (DDSs) is an emerging approach employed to improve the therapeutic effectiveness of a drug (Patra et al., 2018). A DDS can enhance essential drug properties such as solubility and stability in water and may allow for repurposing existing drugs by identifying new applications (Pushpakom et al., 2019; Dinić et al., 2020). This could be the case of old-generation antibiotics, which could take advantage of suitable nanocarriers for overcoming problems associated with resistance phenomena (Kobayashi and Nakazato, 2020). Indeed, the transport of an antibiotic by a nanocarrier might improve the drug bioavailability and pharmacokinetics, change the mechanism of penetration in resistant bacteria with modified walls, and prevent the inactivation by bacterial enzymes and the clearance by efflux pumps as well as further causes of antibiotic resistance (Gupta et al., 2019; Lima et al., 2019; Pham et al., 2019; Eleraky et al., 2020).

**Figure 1:**
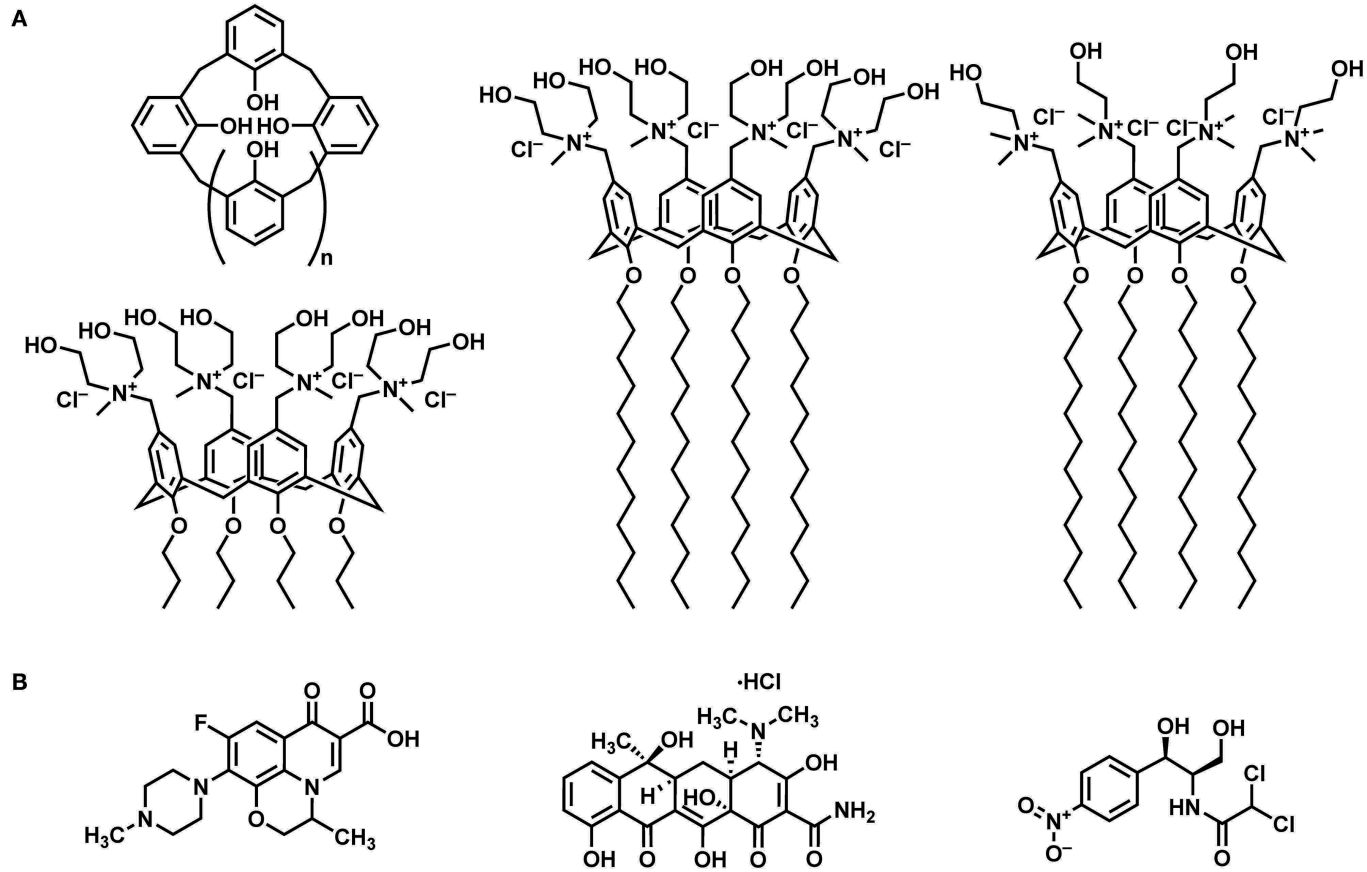
Systems investigated: **(A)** From left to right: schematic representation of a calix[*n*]arene macrocycle, tetra-propoxy-*p*-tetra-(*N,N*-dihydroxyethyl-*N*-methylammonium)-methylene-calix[4]arene (MedeaC4prop), tetra-dodecoxy-*p*-tetra-(*N,N*-dihydroxyethyl-*N*-methylammonium)-methylene-calix[4]arene (MedeaC4dod), and tetra-dodecoxy-*p*-tetra-(*N*,*N*-dimethyl-*N*-hydroxyethylammonium)-methylene-calix[4]arene (CholineC4dod). **(B)** From left to right: ofloxacin, tetracycline hydrochloride, and chloramphenicol.

In the search for novel DDSs, calix[*n*]arene macrocycles, a family of oligomers in which a number *n* of phenolic units are bridged by methylene groups (Figure 1), are attracting great attention. The possibility to functionalize the calixarene upper and lower rim with polar and apolar groups has provided a variety of amphiphilic derivatives with appealing assembly and recognition properties, in which the macrocycle cavity can also act as an additional recognition site. A variety of papers has described the potentialities of cationic or anionic calixarene-based micelles, vesicles, solid lipid nanoparticles, nanocapsules, etc. as DDSs (Lee et al., 2004; Consoli et al., 2018a; Wang et al., 2019).

Polycationic calix[4]arene derivatives that self-assemble in nanoaggregates are promising nanocontainers for delivering antibiotics to bacteria due to their ability to establish electrostatic interactions with the negatively charged bacterial membrane (Formosa et al., 2012). Interestingly, it has been reported that the clustering of cationic groups by a calix[4]arene scaffold (Grare et al., 2007) and the assembly of quaternary ammonium salts in nanoaggregates (Lallemand et al., 2012) result in derivatives with higher antibacterial activity and reduced cytotoxicity to eukaryotic cells when compared with monomeric analogs (Mourer et al., 2009) and other known disinfectants (Grare et al., 2010).

Among the polycationic calix[4]arene amphiphiles, CholineC4dod (Figure 1), bearing choline groups and dodecyl aliphatic chains at the cavity upper and lower rim, respectively, turned out to be a promising nanocarrier for gene (Rodik et al., 2015) and drug delivery (Di Bari et al., 2016a,b). The micellar CholineC4dod system, in the form of colloidal solution (Granata et al., 2017) and hydrogel (Granata et al., 2020), was successfully used for ocular and skin drug delivery in *in vivo* animal models of uveitis and psoriasis (Filippone et al., 2020). Noteworthy, the presence of choline ligands makes CholineC4dod a promising candidate for the vehiculation of antibiotics into bacterial cells. In addition to generating a polycationic surface, the choline moieties could operate as further points of attack for bacterial cell penetration by binding the choline transporters (BCCT, Betaine-Choline-Carnitine Transporter family) present on the surface of bacteria such as *P. aeruginosa* (Lucchesi et al., 1998; Chen et al., 2010; Malek et al., 2011). With this in mind, we decided to investigate whether the amphiphilic CholineC4dod receptor is able to bind target molecules, such as known antibiotics, by non-covalent interactions in aqueous solution.

The determination of the strength and nature of the interactions of a drug with proper carriers, such as micellar assemblies, is essential for the design of novel medicines as well as for the modification or selection of shuttles for target-oriented drug delivery. However, a quantitative analysis of the species, binding affinity, as well as thermodynamic parameters for the recognition/inclusion of drugs in micelles has rarely been addressed (Bouchemal, 2008; Waters et al., 2012; Huang et al., 2020; Kaur et al., 2020).

The present work deals with the study of the binding features of the amphiphilic CholineC4dod receptor with three old-generation antibiotics (Figure 1) in neutral aqueous solution. The interactions of the polycationic derivatives MedeaC4dod and MedeaC4prop (Figure 1) with the same antibiotics were also investigated with the aim of developing new and efficient DDSs.

The examination of the solution equilibria and the determination of species, binding affinity as well as thermodynamic parameters in neutral aqueous solution were carried out using nano-isothermal titration calorimetry (nano-ITC). This is an invaluable technique for determining both stability constant and enthalpy change values for host–guest complex formation (Sgarlata et al., 2009a; Bonaccorso et al., 2012; Giglio et al., 2015) and/or self-organization of surfactants into micelles by a single experiment (Perger and Bešter-Rogač, 2007; De Lisi et al., 2009; Moulik and Mitra, 2009; Loh et al., 2016). Results from calorimetric experiments provided key information on the forces driving the molecular recognition processes involving calixarene-based micellar aggregates and model drugs in water at neutral pH. Dynamic light scattering measurements provided evidences of the nanoaggregate formation whereas mono- and bi-dimensional NMR experiments confirmed the drug–micelle interaction and supported the picture obtained from ITC results on the antibiotic positioning within the calix[4]arene-based micellar backbone. Ofloxacin, tetracycline, and chloramphenicol were selected as models of antibiotics affected by the onset of resistance phenomena with the aim of offering a contribution to the design and development of effective DDSs for the revaluation and use of old-fashioned antibiotics.

## Materials and Methods

### Materials

All chemicals and solvents were purchased from Sigma-Aldrich (Milan, Italy) and used without purification. The hosts tetra-dodecoxy-*p*-tetra-(*N*,*N*-dimethyl-*N*-hydroxyethylammonium)-methylene-calix[4]arene (CholineC4dod) (Rodik et al., 2015, Granata et al., 2017) and tetra-propoxy-*p*-tetra-(*N,N*-dihydroxyethyl-*N*-methylammonium)-methylene-calix[4]arene (MedeaC4prop) (Consoli et al., 2018b) were synthesized as previously reported. The guests (chloramphenicol, ofloxacin, and tetracycline hydrochloride) were purchased from Sigma-Aldrich and used as received. MOPS salt was purchased from Sigma-Aldrich and was of the highest purity commercially available. High-purity water (Millipore, Milli-Q Element A 10 ultrapure water) and A grade glassware were employed throughout.

### Synthesis and Characterization of MedeaC4dod

A solution of *N*-methyldiethanolamine (26 mg, 218 μmol) in THF (0.4 ml) was added to a stirring solution of tetra-dodecoxy-*p*-chloromethyl calix[4]arene (52 mg, 40 μmol) in THF (1.5 ml). The reaction mixture was refluxed for 24 h. After cooling, the suspension was diluted with THF (5 ml) and diethyl ether (15 ml) and centrifuged (4,000 rpm, 5 min). The precipitate was washed with a mixture of THF (2 ml) and diethyl ether (8 ml) and then with diethyl ether (3 × 5 ml) by repeated centrifugation (4,000 rpm, 5 min) and removal of the supernatant. The solid was dried under vacuum for 24 h to give a white powder (61 mg, 86% yield). NMR spectra were recorded on a Bruker 400-mHz spectrometer equipped with a 5-mm inverse detection gradient probe. Chemical shifts (δ, ppm) are relative to the residual proton solvent peak; coupling constant (*J*) values are given in Hz. ^1^H-NMR (MeOD): δ 0.89 (t, 12H, *J* = 7.2 Hz, 4 × CH_3_), 1.31 (br m, 64H, 32 × CH_2_), 1.46 (br m, 8H, 4 × CH_2_), 2.00 (br m, 8H, 4 × CH_2_), 2.95 (s, 12H, 4 × NCH_3_), 3.30–3.45 (overlapped, 12H, 2 × ArCH_2_Ar and 4 × CH_2_N), 3.49 (br t, 8H, 4 × CH_2_N), 3.88 (t, *J* = 7.2 Hz, 8H, 4 × OCH_2_), 3.90–4.10 (overlapped, 16H, 8 × CH_2_OH), 4.45–4.56 (overlapped, 12H, 2 × ArCH_2_Ar and 4 × ArCH_2_N), 7.05 (s, 8H, 8 × ArH). ^13^C-NMR (MeOD): δ 14.5 (q), 23.8, 27.8, 30.7, 31.0, 31.1, 31.2, 31.4, 31.8, 33.2 (t), 49.4 (q), 56.5, 56.8, 58.9, 64.2, 68.9, 77.0 (t), 122.9 (d), 135.1, 136.9, 159.7 (s). ESI-HRMS spectra were acquired on a Thermo Scientific Exactive Plus Orbitrap MS (source voltage 3.5 kV; capillary voltage 82.5 V; tube lens voltage 150 V). HR ESI-MS: (m/z) calcd for C_100_H_176_Cl_3_N_4_${\text{O}}_{12}^{+}$ [M-Cl^−^]^+^ = 1732.2316, found [M-Cl^−^]^+^ = 1732.2292; calcd for C_100_H_176_Cl_2_N_4_${\text{O}}_{12}^{2+}$ [M-2Cl^−^]^2+^ = 848.6311, found [M-2Cl^−^]^2+^ = 848.6317.

### Dynamic Light Scattering (DLS)

The samples were prepared by dissolution of CholineC4dod, MedeaC4dod, and MedeaC4prop (0.2 mM) in 10 mM MOPS buffer (pH 7.2). Size measurements were performed on a ZetaSizer Nano ZS90 Malvern Instrument (UK), equipped with a 633-nm laser, at a scattering angle of 90° and at 25°C. Each measurement was performed three times.

### ITC Titrations

ITC titrations were carried out at 25°C with a nano-isothermal titration calorimeter (nano-ITC, TA Instruments) having an active cell volume of 988 μl and equipped with a 250-μl injection syringe. The reaction mixture in the sample cell was stirred at 250 rpm during the titration. Measurements were run in the overfilled mode to prevent possible issues from liquid evaporation or the presence of the vapor phase (Bundle and Sigurskjold, 1994; Hansen et al., 2011).

The power curve was integrated by using the NanoAnalyze software (TA Instruments) to obtain the gross heat evolved/absorbed in the reaction. The calorimeter was calibrated chemically by a test reaction (HCl/TRIS) according to the procedure previously described (Sgarlata et al., 2013). An electrical calibration of the instruments was also performed. All solutions were degassed with gentle stirring under vacuum for about 15 min before each titration experiment.

ITC measurements for the study of the interactions of the guests with the micellar aggregates were carried out titrating a solution of tetracycline (2 ÷ 2.5 mM), ofloxacin (2 ÷ 2.5 mM), or chloramphenicol (from 2 to 10 mM) into a CholineC4dod or MedeaC4dod solution (0.2 mM) in the micellar/aggregated form. The same conditions were used for the titrations of the antibiotics with MedeaC4prop host. Further experiments were carried out titrating a solution of tetracycline (2 ÷ 2.5 mM), ofloxacin (2 ÷ 2.5 mM), or chloramphenicol (from 2 to 10 mM) into a solution of CholineC4dod or MedeaC4dod in their monomeric form (2.5 μM).

Both host and guest solutions were prepared at pH 7.2, dissolving proper amounts of the compounds in 10 mM MOPS, in order to reproduce conditions for neutral pH aqueous solutions and minimize any heat contribution resulting from the interaction of the compounds with the proton.

Typically, at least three independent titrations were run for each host–guest system in order to collect a suitable number of points to obtain a satisfactory fit of the calorimetric curves. The heats of dilution were determined in separate blank experiments titrating solutions of each guest (prepared in MOPS buffer) into a solution containing MOPS buffer only.

The net heats of reaction, obtained by subtracting the gross heat by that evolved/absorbed in the blank experiments, were analyzed by HypCal (Arena et al., 2016). This software allows for the simultaneous determination of standard enthalpy and binding constant values and has been specifically designed for the treatment of data obtained from ITC instruments operating in overfilled mode. The thermodynamic parameters were obtained by optimizing the agreement between observed and calculated reaction heats. The optimization is performed by a non-linear least squares analysis, minimizing the objective function (*U*):

(1) $$U=\sum {\left(Q_{obs.}-Q_{calc.}\right)}^{2}$$

where *Q*_*obs*_ is the observed heat for a given reaction step, corrected for the dilution (blank) effects, while *Q*_*calc*_ is calculated as:

(2) $$\begin{array}{l} Q_{calc.}=-\sum \delta n\Delta H^{0} \end{array}$$

where δ*n* is the change in the number of moles of a reaction product and Δ*H*^0^ is the molar formation enthalpy of the reaction product. The sum is carried out over all the reaction steps; the squared residuals (*Q*_*obs*_ –* Q*_*calc*._)^2^ are summed over all the titration points. Stability constant values and thermodynamic parameters were obtained analyzing simultaneously calorimetric data obtained from different titrations.

### NMR Analysis

The samples were prepared by addition of chloramphenicol, ofloxacin, or tetracycline hydrochloride (2.1 mM) to a solution of CholineC4dod (1.4 mM) in MOPS/D_2_O (10 mM). All 1D- and 2D-NMR spectra were acquired on a Bruker Avance 400 spectrometer (^1^H NMR 400.13 MHz) at 297 K. Chemical shifts (δ) are expressed in parts per million (ppm), referenced to the residual proton water peak. The proton spectra were recorded with a water suppression program. In 2D-NOESY experiments, the mixing time was 360 ms with 2 s of recycle delay. Data were processed using the TopSpin 2.1 software (Bruker).

## Results and Discussion

### Calix[4]arene Derivatives Preparation and Characterization

**Figure 2:**
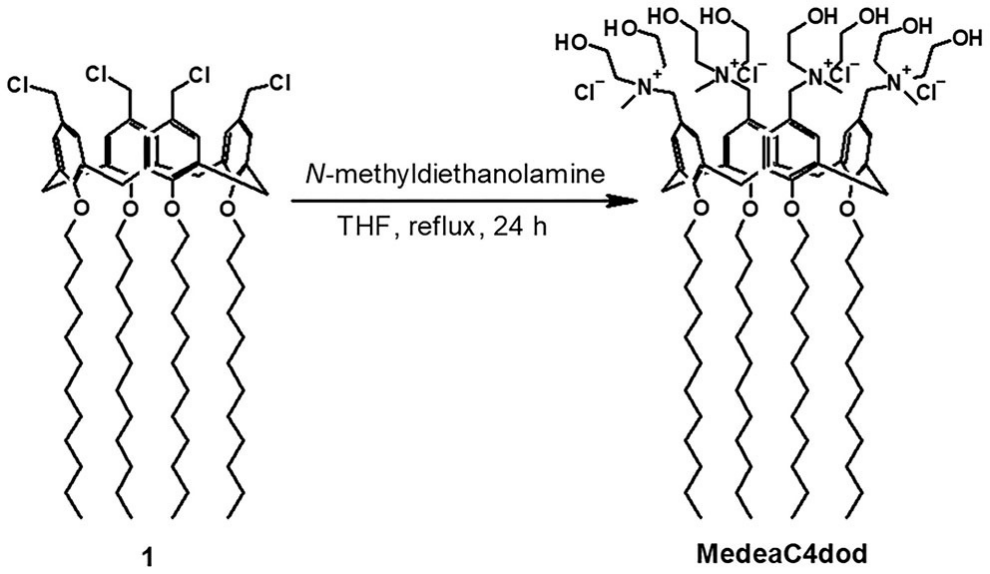
Synthesis of MedeaC4dod from the precursor **1**.

CholineC4dod (Rodik et al., 2015; Granata et al., 2017) and MedeaC4prop (Consoli et al., 2018b) were prepared and characterized as previously reported while MedeaC4dod was prepared by adapting the same synthetic procedure. Tetra-dodecoxy-chloromethyl-calix[4]arene derivative **1** reacted with *N*-methyldiethanolamine in THF (Figure 2) to give MedeaC4dod in high yield (86%). MedeaC4dod was characterized by high-resolution ESI-MS and 1D- and 2D-NMR spectra (Supplementary Figures 1–4) that clearly indicated the exhaustive tetra-functionalization of the calix[4]arene upper rim. The mass spectrum showed two ion peaks at 1732.23 and 848.63 relative to [M-Cl^−^]^+^ and [M-2Cl^−^]^2+^, respectively. The presence of one AX system for the ArCH_2_Ar groups of the macrocycle in the proton NMR spectrum was consistent with a *cone* conformation (Gutsche, 1989) and a fully symmetric structure, corroborated by the resonances of the *N*-methyldiethanolammonium substituents.

Dynamic light scattering measurements showed that, in MOPS buffer (10 mM, pH 7.2), CholineC4dod and MedeaC4dod (0.2 mM) form nanoaggregates with mean hydrodynamic diameters of 7.0 and 7.5 nm, respectively (Supplementary Figures 5, 6). The length and dimensions of a single calixarene lead to the conclusion that these size values are consistent with the formation of a micellar-like structure. No well-defined aggregates were instead observed at the same concentration for MedeaC4prop, which bears shorter alkyl chains.

### Solution Thermodynamics of the Drug–Micelle Systems

Depending on the type and site of interaction, a guest can arrange with a micellar system in different ways. It may be completely incorporated in the hydrophobic core by selective interaction with the aliphatic or bulkier chains, may penetrate up to a certain depth, or may be adsorbed on the micellar surface. Nano-calorimetric experiments were carried out to (1) evaluate whether selected model antibiotics are able to effectively interact with micellar aggregates formed by the polycationic amphiphilic calixarenes and (2) determine the binding parameters and driving forces for the molecular recognition equilibria involving drugs and micelles in aqueous solution at neutral pH.

The ITC study was carried out by titrating solutions of tetracycline, ofloxacin, and chloramphenicol into MedeaC4dod or CholineC4dod solutions at 0.2 mM concentration, in order to have the micellar aggregate into the calorimetric vessel at the tested conditions (Di Bari et al., 2016b).

A typical ITC titration for the tetracycline–CholineC4dod system in neutral aqueous solution (pH 7.2, MOPS) at 25°C is shown in Figure 3. ITC titration curves for the other drug-calixarene systems are shown in Supplementary Figures 7–11 together with the corresponding blank experiments (Supplementary Figures 12–14).

**Figure 3:**
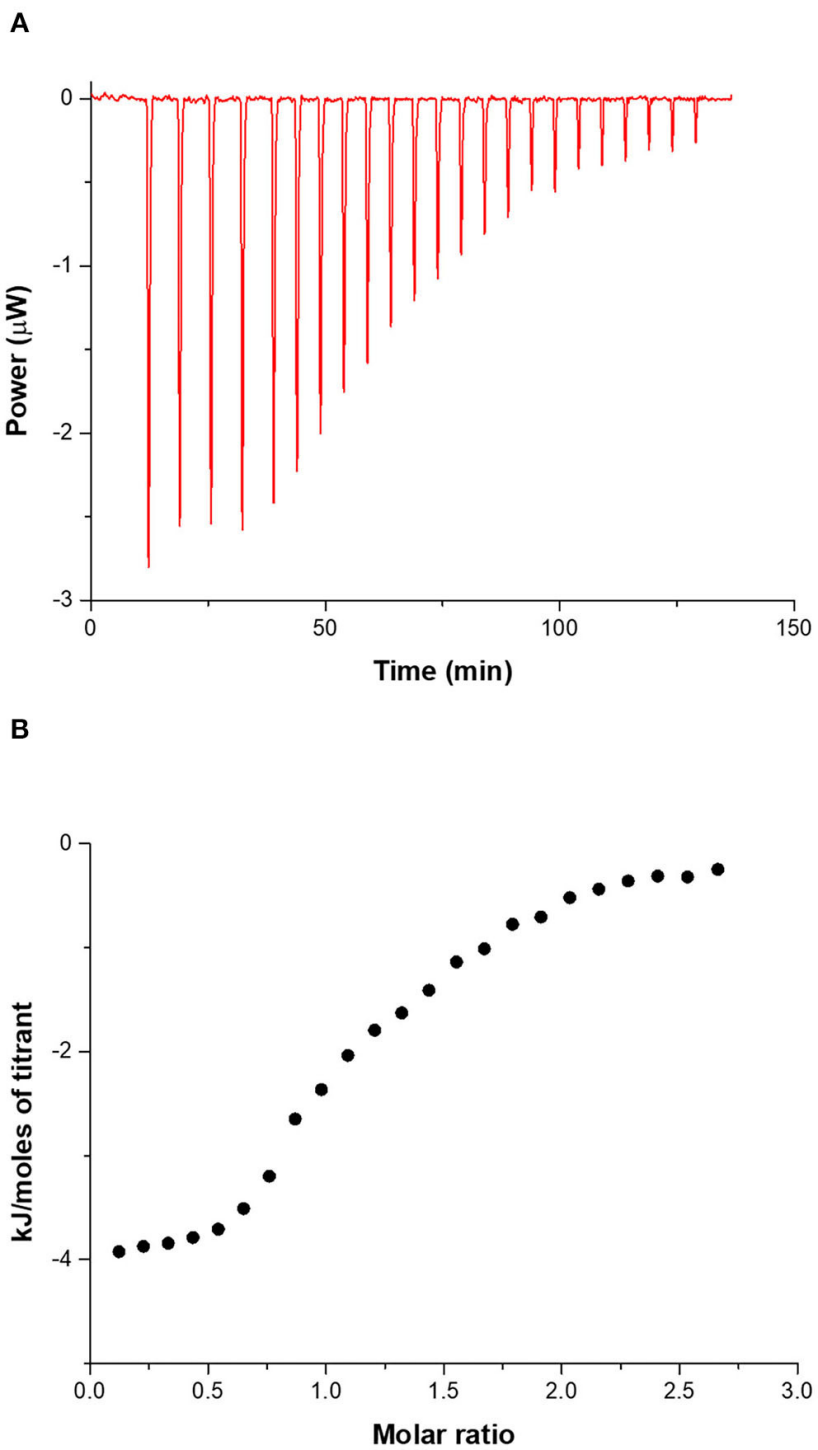
**(A)** ITC titration of tetracycline 2 mM into CholineC4dod 0.2 mM at 25°C in neutral aqueous solution (pH 7.2, MOPS). **(B)** Integrated heat data.

The curves clearly show that the net heat released/adsorbed when the antibiotics are titrated into a micellar calixarene solutions is remarkable (i.e., the gross reaction heat is significantly larger than the heat from blank experiments) and the integrated heat data exhibit a pattern that may be analyzed for obtaining thermodynamic parameters. Interestingly, the interaction of chloramphenicol with both the calixarene micellar systems is exothermic (Supplementary Figures 10, 11) while the reaction of the other two drugs with the same systems are endothermic, thus indicating that different driving forces are involved in the recognition process of these drugs in solution.

To assess whether the guest molecules are able to interact with the calixarene receptor regardless of its form (i.e., monomer vs. micellar aggregate) and hence to explore whether the formation of micelles is required for guest recognition, ITC measurements were also carried out titrating the drugs into MedeaC4dod or CholineC4dod solutions at a 500-fold lower concentration (2.5 μM) in order to deal only with the monomeric form. The overlap between blank experiments and titrations (Supplementary Figures 15–20) unambiguously revealed that, at this concentration, both the calixarenes are not capable of establishing any significant interaction with tetracycline, ofloxacin, and chloramphenicol as no detectable net heat is released/absorbed upon host–guest titration. This result highlights the key role played by the micellar assembly: the effective recognition/entrapment of the target guest cannot occur unless multiple host molecules are suitably aggregated to form a micellar-like arrangement.

ITC titrations were also carried out, at the same experimental conditions employed for the micellar aggregates, using MedeaC4prop, which has the same upper rim of MedeaC4dod but shorter alkyl substituents at the lower rim and, consequently, is not able to form micelles at the tested concentration (0.2 mM). The aim of these experiments is to (1) define the portion or rim of calixarene backbone actually involved in the interaction/recognition of the model drugs and (2) examine whether the guest is included into the calixarene cavity. The negligible heat values recorded in these ITC experiments (Supplementary Figure 21), the shape of the calorimetric curves (Supplementary Figure 22), and the basically full overlap between the heat rate from blank experiments and host–guest titrations (Supplementary Figure 23) indicated that no detectable reaction occurred in the calorimetric vessel upon titration and thus no binding interactions are observed in the presence of the non-aggregating MedeaC4prop host. Overall, these evidences proved that the guests are not included into the host cavity and do not interact with the four *N*-methyldiethanolammonium groups at the upper rim of the macrocyclic receptor in solution. While it may be expected that the charged groups at the upper rim cause a steric encumbrance that prevent the guest to enter the cavity, the lack of heat recorded upon titration allows one to rule out also other kinds of interactions between the guest and the calixarene polar head groups.

Since it was observed that all guests are not able to enter or interact with the host cavity and with the long alkyl tails at the lower rim when the calixarene is in its monomeric form, it may be concluded that the cooperative effect generated by the assembling in micelles is the boost for the efficient drug entrapment/interaction by both MedeaC4dod and CholineC4dod.

Data obtained by ITC measurements were analyzed using a model that assumes the formation of a 1:1 species between the guest and the micellar aggregate, in line with many research groups that employ the “one site” binding model for the refinement of the thermodynamic parameters of these systems (Maity et al., 2015; Banipal et al., 2017).

**Figure 4:**
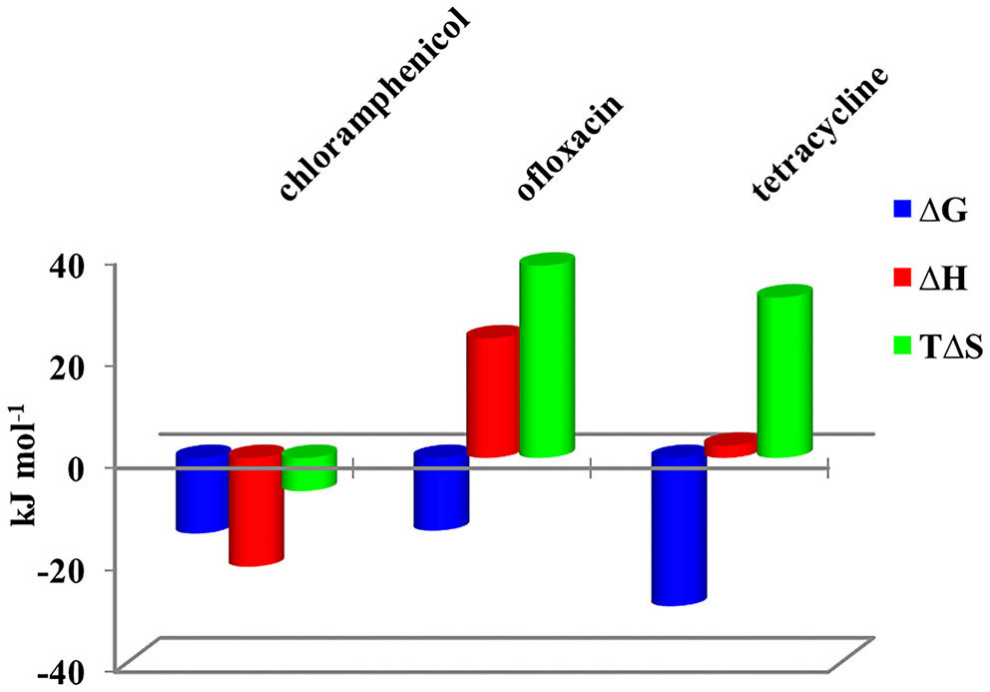
Thermodynamic parameters for the complex formation of CholineC4dod-based micelles with model drugs at 25°C in neutral aqueous solution.

**Figure 5:**
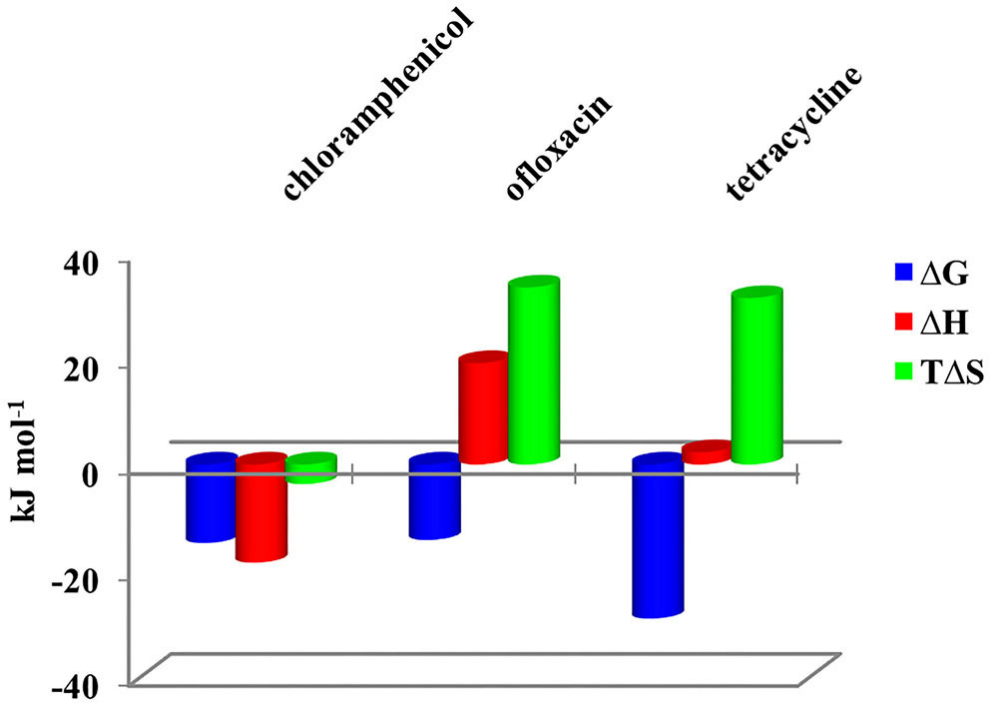
Thermodynamic parameters for the complex formation of MedeaC4dod-based micelles with model drugs at 25°C in neutral aqueous solution.

Calorimetric data were analyzed by HypCal (Arena et al., 2016), a software designed for the determination of binding affinity and Δ*H* values for the formation of host–guest or micelle–guest adducts. Multiple titrations were simultaneously refined by the program. A typical HypCal output for the adducts formed by tetracycline with the micellar aggregate based on CholineC4dod is shown in Supplementary Figure 24; for each titration, the overlap between observed and calculated values is displayed. The binding constants and the thermodynamic parameters for the micelle–guest complex formation are reported in Tables 1, 2 and shown in Figures 4, 5.

**Table 1:** Log*K* values and thermodynamic parameters for the interaction of CholineC4dod-based micelles and model drugs at 25°C in neutral aqueous solution (pH 7.2, MOPS buffer).

**Guest**	**Log*K***	**Δ*H* (kJ mol^**−1**^)**	**Δ*S* (J mol^**1**^ deg^**−1**^)**
Tetracycline	5.1 (3)	2.41 (4)	107 (6)
Ofloxacin	2.5 (1)	23.5 (1)	127 (2)
Chloramphenicol	2.6 (1)	−21.35 (3)	−21.5 (9)

*σ in parentheses*.

**Table 2:** Log*K* values and thermodynamic parameters for the interaction of MedeaC4dod-based micelles and model drugs at 25°C in neutral aqueous solution (pH 7.2, MOPS buffer).

**Guest**	**Log*K***	**Δ*H* (kJ mol^**−1**^)**	**Δ*S* (J mol^**−1**^ deg^**−1**^)**
Tetracycline	5.1 (3)	2.31 (4)	105 (7)
Ofloxacin	2.6 (1)	19.2 (1)	114 (2)
Chloramphenicol	2.6 (1)	−18.55 (3)	−11.7 (9)

*σ in parentheses*.

Data reported in Tables 1, 2 show no relevant difference in the binding parameters for CholineC4dod and MedeaC4dod as the two calixarenes, although bearing a different polar head, have the same C12 aliphatic chains, which are functional to the micelle formation.

The binding affinity values for the adduct formation of the micellar aggregates with chloramphenicol and ofloxacin are similar, but the splitting of Δ*G* into Δ*H* and Δ*S* values enabled the highlighting of factors and forces that are not expressed in the free Gibbs energy value. Indeed, the binding of chloramphenicol to the calixarene-based micelles is enthalpically favored and driven (|Δ*H*| > |*T*Δ*S*|) but entropically unfavored (Sgarlata et al., 2009b; Bonaccorso et al., 2012, 2017). The favorable enthalpic contribution may be due to the insertion of chloramphenicol within the micelle palisade layer by establishing CH–π, ion–π, and van der Waals interactions between the calixarenes and the aromatic ring and/or the nitro group of chloramphenicol (Choudhary et al., 2015; Mukhija and Kishore, 2017). This favorable enthalpic contribution could also be attributed to “frustrated” water molecules that leave the micelle core and create a new hydrogen bonding network with the bulk water molecules and the micelle surface.

Conversely, the interaction of ofloxacin with both calixarene-based micelles is an entropy-driven and favored (|Δ*H*| < |*T*Δ*S*|) but enthalpy unfavored process. The entropic contribution is due to the desolvation of the guest as well as of the micelle surface upon guest binding to the micellar aggregate. These results suggest that ofloxacin should not be able to insert into the palisade layer of the micelles while (weakly) interacting with the positively charged exterior surface of the micelles. These interactions with the surface of the aggregates cause the release of water of hydration to the bulk solvent (large and positive entropy values). The enthalpic cost for desolvation (bonds breaking) results in a positive Δ*H* value.

The interaction of tetracycline with both the calixarene-based micelles is an entropy-driven and favorite (|Δ*H*| < |*T*Δ*S*|) but enthalpy unfavored process, as already observed for ofloxacin. However, despite the fact that the general trend of the thermodynamic parameters is similar for these two antibiotics, Δ*H*_tetracycline_ < Δ*H*_ofloxacin_ (2.41 vs. 23.50 kJ mol^−1^), the binding affinity of tetracycline to the micelles is larger (log*K*_tetracycline_ = 5.1 vs. log*K*_ofloxacin_ = 2.5). The less unfavorable Δ*H* value observed for tetracycline, which eventually leads to a larger affinity for the micelles in solution, is probably due to cation–π interactions between the calixarene hydrophilic head and the aromatic rings of the tetracycline backbone, which advantageously balance the enthalpic cost for desolvation. As in the case of ofloxacin, the large and favorable entropic contribution is due to the release of water molecules from both the guest and the calixarene micellar aggregates (Choudhary et al., 2015; Mukhija and Kishore, 2017).

### NMR Characterization of the Drug–Micelle Systems

NMR is one of the techniques used to gain deeper insight into micelle–drug interactions (Wong, 2006). Due to the accuracy and precision of a NMR spectrometer, a change of 0.01 ppm or greater in the proton resonance is considered a significant change. Thus, to confirm the binding features and interactions of the micellar calixarenes with the antibiotics and support the ITC results, we recorded mono- and bi-dimensional proton spectra of CholineC4dod in combination with chloramphenicol, ofloxacin, or tetracycline in neutral aqueous solution (MOPS/D_2_O).

**Figure 6:**
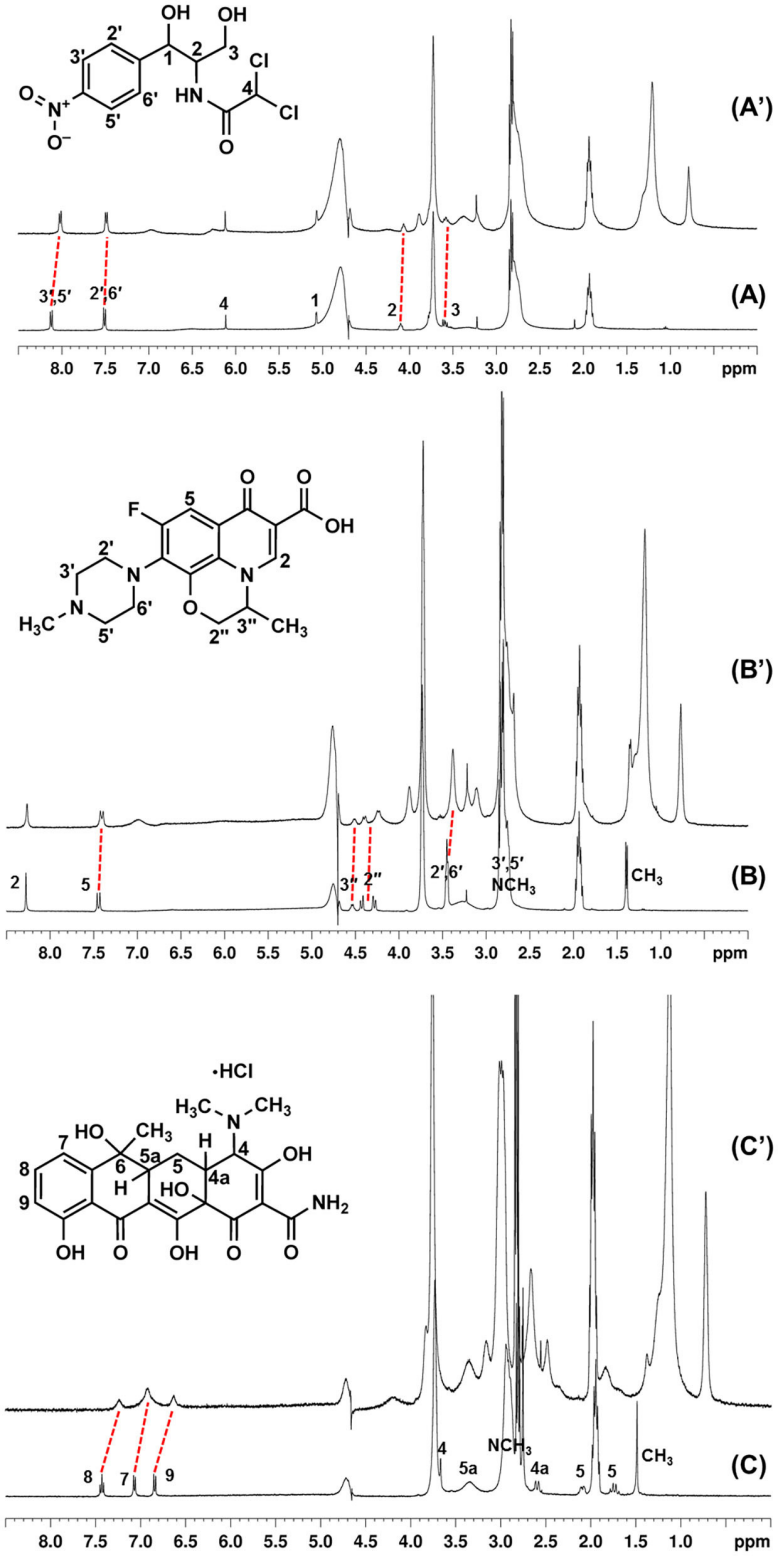
^1^H-NMR spectra of chloramphenicol **(A)**, ofloxacin **(B)**, and tetracycline **(C)** alone and in the presence of CholineC4dod (**A'**, **B'** and **C'** respectively), pD 7.4 (MOPS buffer/D_2_O), 297 K.

The proton signals of chloramphenicol in the presence of the micellar CholineC4dod (Figure 6) showed an upfield shift of 0.106 ppm and 0.024 ppm for ArH (3′,5′and 2′,6′respectively), 0.036 ppm for CH-N (2), and 0.014 ppm for CH_2_OH (3). These evidences could be indicative of CH–π, ion–π, and van der Waals interactions between the calixarene and the aromatic ring and/or the nitro group of the chloramphenicol, in agreement with the higher upfield shift of the chloramphenicol ArH (3′,5′) near the nitro group. Nuclear Overhauser effect (NOESY) correlations between the chloramphenicol aromatic protons and the CH_2_ protons of the dodecyl chains at the calix[4]arene lower rim (Supplementary Figure 25) corroborated the insertion of the guest within the micelle palisade layer suggested by ITC analysis.

The interaction of ofloxacin with the micellar CholineC4dod was supported by the upfield shift of the antibiotic ArH (5, 0.034 ppm), N-CH_2_ (2′,6′, 0.065 ppm), N-CH (3′′, 0.02 ppm), O-CH_2_ (2′′, 0.026 and 0.044 ppm), and C-CH_3_ (0.037 ppm) protons (Figure 6). In NOESY experiments, the overlap of the ofloxacin N-CH_2_ (3′,5′) and N-CH_3_ signals with the resonances of CholineC4dod N-CH_3_ and MOPS buffering agent made difficult the exact signal assignment. Nevertheless, if we attribute the observed NOESY signals, which are absent in the spectra recorded with the other antibiotics, to the ofloxacin protons (Supplementary Figure 26), the correlations with the calix[4]arene ArH, ArCH_2_Ar, CH_2_N and CH_2_OH/ArOCH_2_ (overlapped) groups suggest that ofloxacin lies in a more external region of the micelle if compared to chloramphenicol. Beside cation–π and CH–π interactions, the electron-rich fluorine atom and the carboxyl group of the ofloxacin could establish ion–dipole and ion–ion interactions with the nitrogen atoms of CholineC4dod.

Overlapping phenomena made also difficult the assignment/detection of some tetracycline signals in the drug–micelle NMR spectra. Nevertheless, the clear-cut upfield shift of the tetracycline aromatic protons (0.19, 0.15, and 0.21 ppm for the protons in positions 8, 7, and 9, respectively) and C-CH_3_ protons (0.059 ppm) compared to the free tetracycline (Figure 6) suggested the existence of interactions between the calixarene hydrophilic heads and the aromatic rings on the tetracycline backbone in line with ITC evidences. The higher shift of the tetracycline proton in position 9 could also be ascribable to interactions between the OH group of tetracycline and the choline heads in the micelle surface. These data indicate a more superficial positioning of tetracycline in the micelle–drug adduct when compared to the other two antibiotics.

## Conclusions

Calixarene-based micelles are promising DDSs and the assessment of what forces drive the drug recognition processes is a fundamental step to the design and development of increasingly efficient nanocarriers. Since there is a strong demand for antibiotic delivery systems that could be valid allies in the fight against antibiotic-resistant bacteria, which are a serious threat to the health of all living beings, we investigated the interaction of three old-generation antibiotics with polycationic calix[4]arene amphiphiles in neutral aqueous solutions.

At the tested conditions, the antibiotics successfully interacted only with the calix[4]arene derivatives able to form micellar aggregates evidencing that micelle formation is crucial for the guest recognition process to occur in solution. ITC measurements showed that the formation of the chloramphenicol–micelle adduct is always an enthalpically driven process while the adducts with ofloxacin and tetracycline are always entropically driven and enthalpically unfavored. Combined ITC and NMR analysis suggested a different location of the three antibiotics in the micelle backbone depending on their structure, charge, and functional groups.

## Data Availability Statement

The raw data supporting the conclusions of this article will be made available by the authors, without undue reservation.

## Author Contributions

CS and GC: conceptualization, methodology, supervision, and writing—review and editing. RM, GG, and AR: investigation, methodology, and formal analysis. RM, CS, and GC: writing—original draft preparation. All authors: contributed to the article and approved the submitted version.

## Conflict of Interest

The authors declare that the research was conducted in the absence of any commercial or financial relationships that could be construed as a potential conflict of interest. The handling editor declared a past co-authorship with one of the author CS.
