# Supplementary material for: Binding Affinity and Driving Forces for the Interaction of Calixarene-Based Micellar Aggregates With Model Antibiotics in Neutral Aqueous Solution
Source: Front Chem. 2021 Jan 14;8:626467. doi: 10.3389/fchem.2020.626467 (PMC7841070; doi:10.3389/fchem.2020.626467)
Supplement: Supplementary file 1 [file Data_Sheet_1.PDF]

*Supplementary Material*

# Binding affinity and driving forces for the interaction of calixarene-based micellar aggregates with model antibiotics in neutral aqueous solution

**Rossella Migliore[1], Giuseppe Granata[2], Andrea Rivoli[1], Grazia Maria Letizia Consoli[2*], Carmelo Sgarlata[1*]**

[1] Dipartimento di Scienze Chimiche, Università degli Studi di Catania, Viale Andrea Doria 6, 95125, Catania, Italy

[2] Istituto di Chimica Biomolecolare, CNR, Via P. Gaifami 18, 95126 Catania, Italy

***Correspondence:**
Carmelo Sgarlata, Grazia Maria Letizia Consoli
sgarlata@unict.it; grazia.consoli@icb.cnr.it

## Table of content

**Figure S1.** $^{1}$H NMR spectrum of MedeaC4dod. S3

**Figure S2.** $^{13}$C NMR spectrum of MedeaC4dod. S3

**Figure S3.** 2D-COSY NMR spectrum of MedeaC4dod. S4

**Figure S4.** 2D-HSQC NMR spectrum of MedeaC4dod. S4

**Figure S5.** DLS of CholineC4dod. S5

**Figure S6.** DLS of MedeaC4dod. S5

**Figure S7.** ITC titration of tetracycline into MedeaC4dod. S6

**Figure S8.** ITC titration of ofloxacin into MedeaC4dod. S6

**Figure S9.** ITC titration of ofloxacin into CholineC4dod. S7

**Figure S10.** ITC titration of chloramphenicol into MedeaC4dod. S7

**Figure S11.** ITC titration of chloramphenicol into CholineC4dod. S8

**Figure S12.** ITC titration of tetracycline into MOPS. S8

**Figure S13.** ITC titration of ofloxacin into MOPS. S9

**Figure S14.** ITC titration of chloramphenicol into MOPS. S9

**Figure S15.** ITC titration and blank experiment for the tetracycline/MedeaC4dod system. S10

**Figure S16.** ITC titration and blank experiment for the tetracycline/CholineC4dod. S10

**Figure S17.** ITC titration and blank experiment for the ofloxacin/MedeaC4dod system. S11

**Figure S18.** ITC titration and blank experiment for the ofloxacin/CholineC4dod system. S11

**Figure S19.** ITC titration and blank experiment for the chloramphenicol/MedeaC4dod system. S12

**Figure S20.** ITC titration and blank experiment for the chloramphenicol/CholineC4dod system. S12

**Figure S21.** Net heat curve for the tetracycline/ MedeaC4prop system. S13

**Figure S22.** ITC titration of ofloxacin into MedeaC4prop. S13

**Figure S23**. ITC titration and blank experiment for the chloramphenicol/MedeaC4prop system. S14

**Figure S24**. HypCal output. S14

**Figure S25.** 2D-NOESY NMR spectrum of CholineC4dod with chloramphenicol. S15

**Figure S26.** 2D-NOESY NMR spectrum of MedeaC4dod with ofloxacin. S16

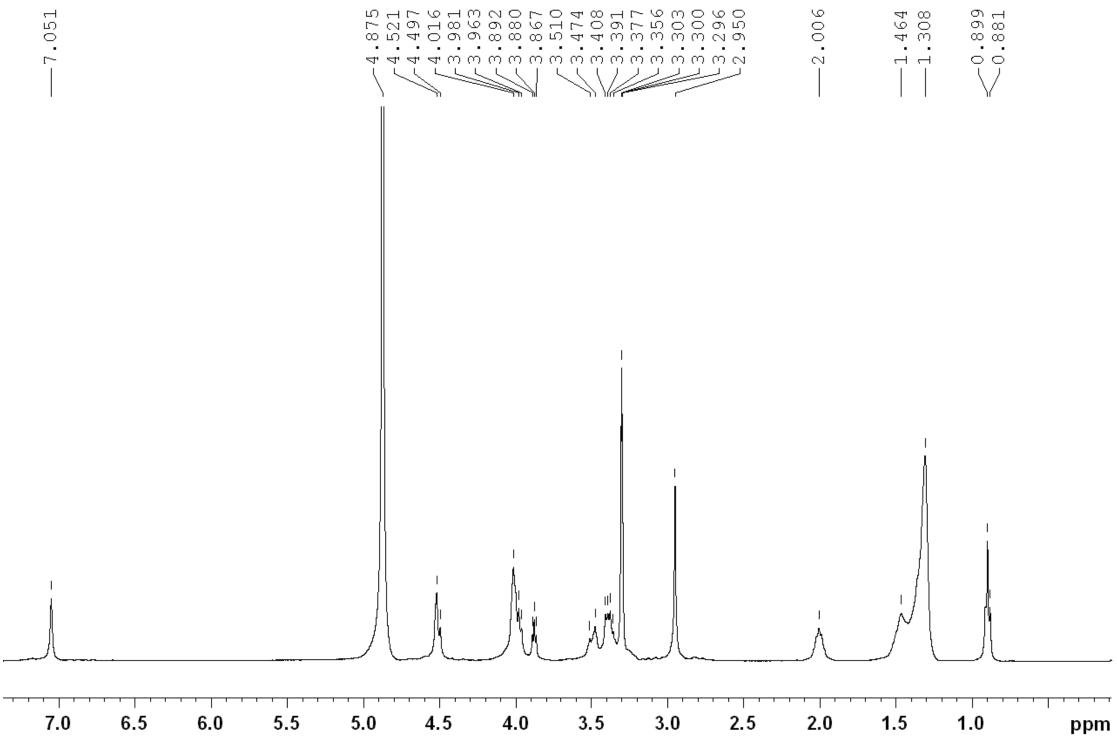


**Figure S1.** $^{1}$H NMR spectrum of MedeaC4dod ($CD_3OD$, 297 K).

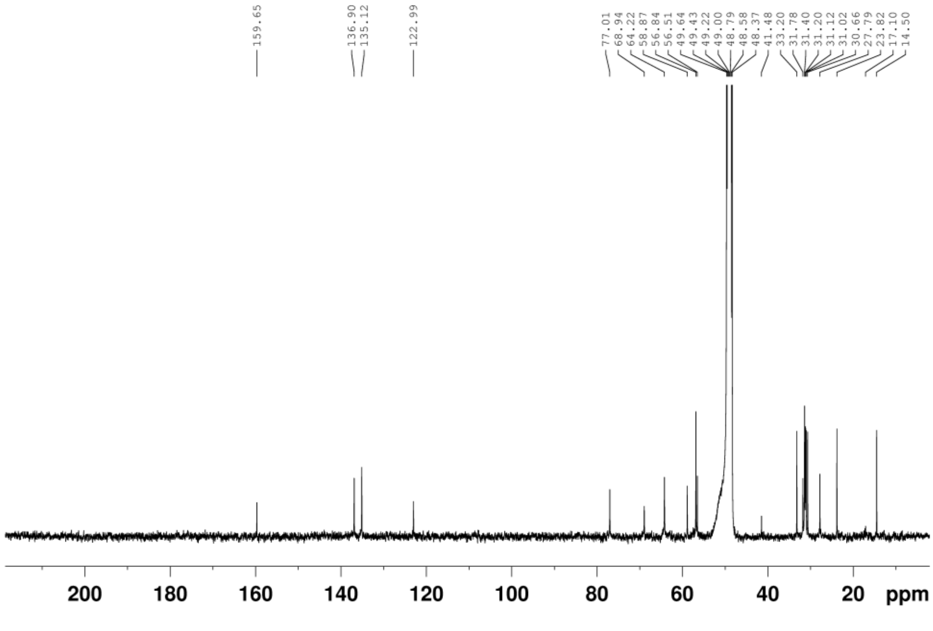


**Figure S2.** $^{13}$C NMR spectrum of MedeaC4dod ($CD_3OD$, 297 K).

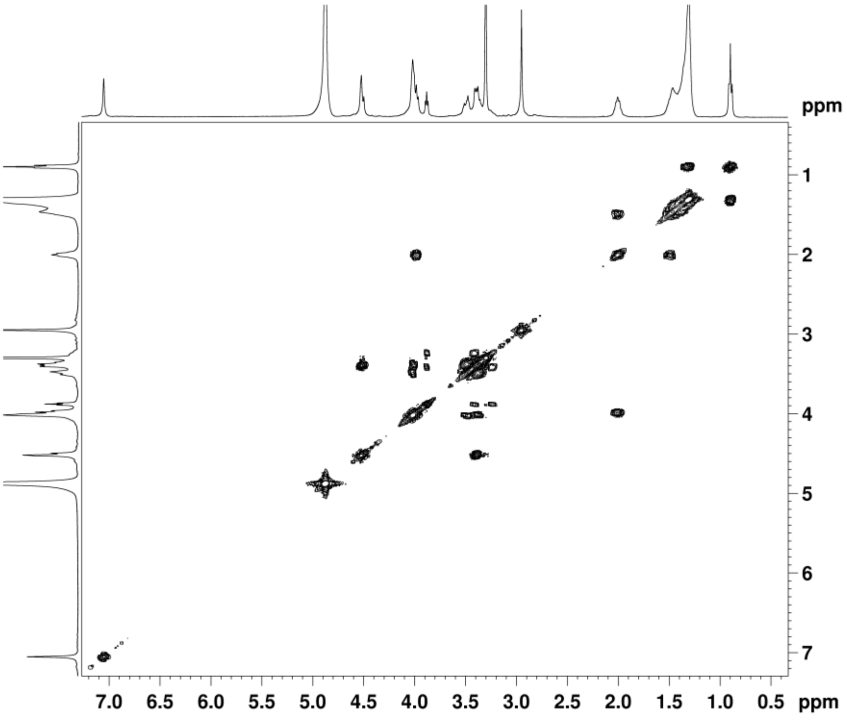


**Figure S3.** 2D-COSY NMR spectrum of MedeaC4dod ($CD_3OD$, 297 K).

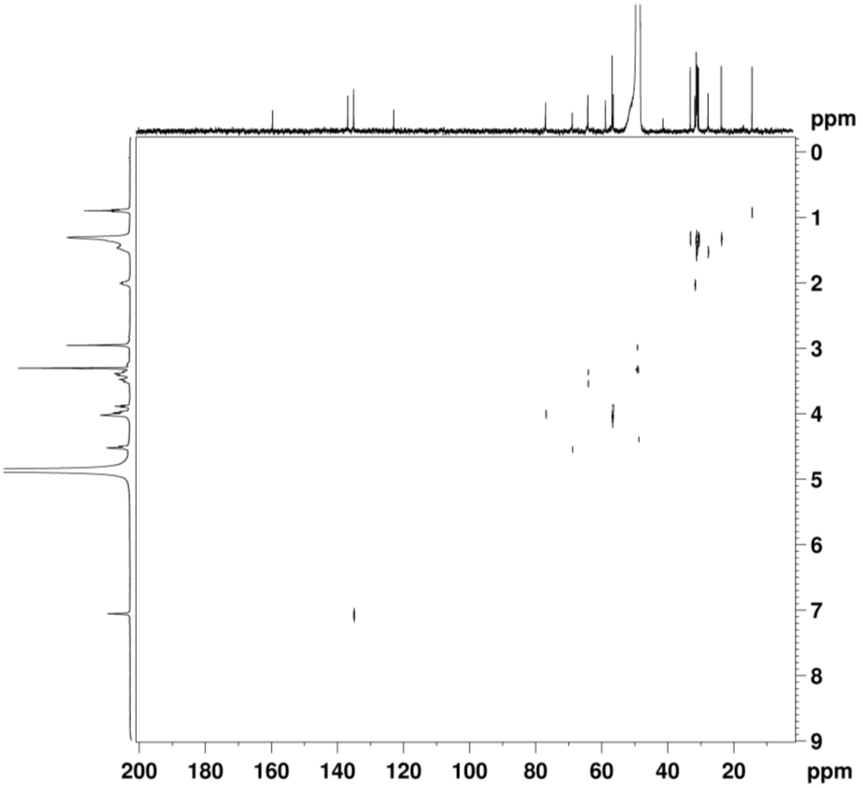


**Figure S4.** 2D-HSQC NMR spectrum of MedeaC4dod ($CD_3OD$, 297 K).

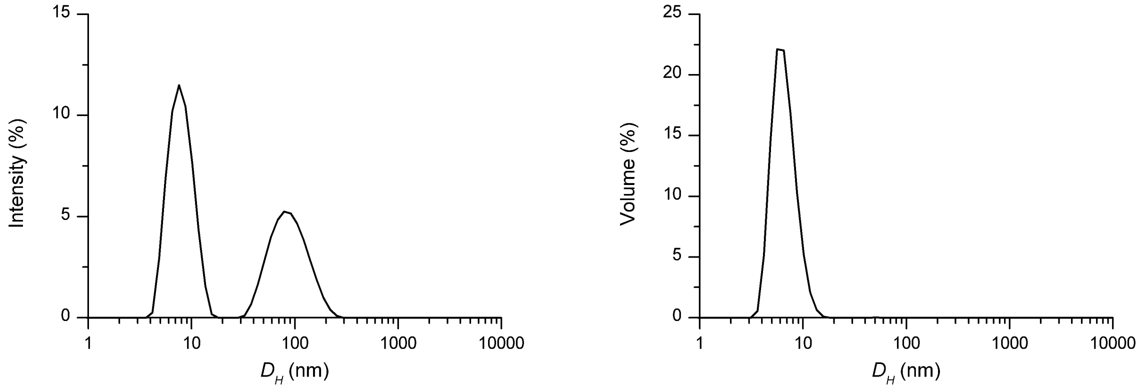


**Figure S5.** DLS of CholineC4dod (0.2 mM): distribution size in I % (left) and V % (right). The graph in V % evidenced a higher number of smaller nanoaggregates.

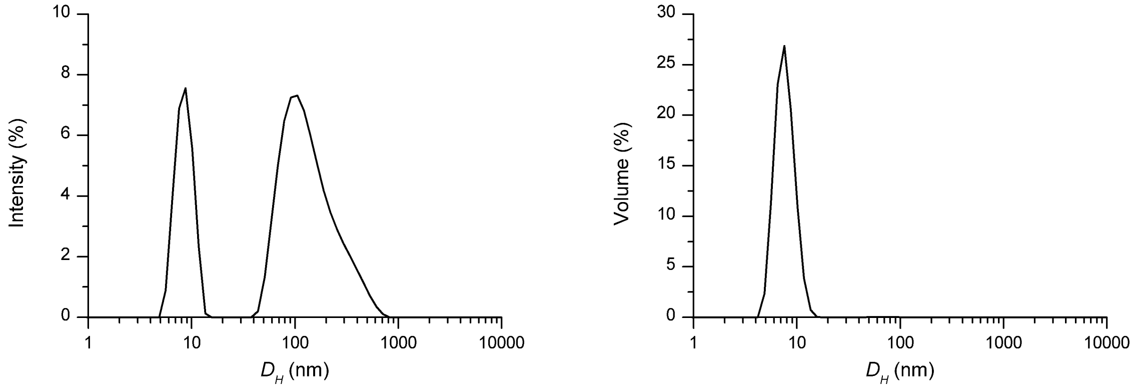


**Figure S6.** DLS of MedeaC4dod (0.2 mM): distribution size in I % (left) and V % (right). The graph in V % evidenced a higher number of smaller nanoaggregates.

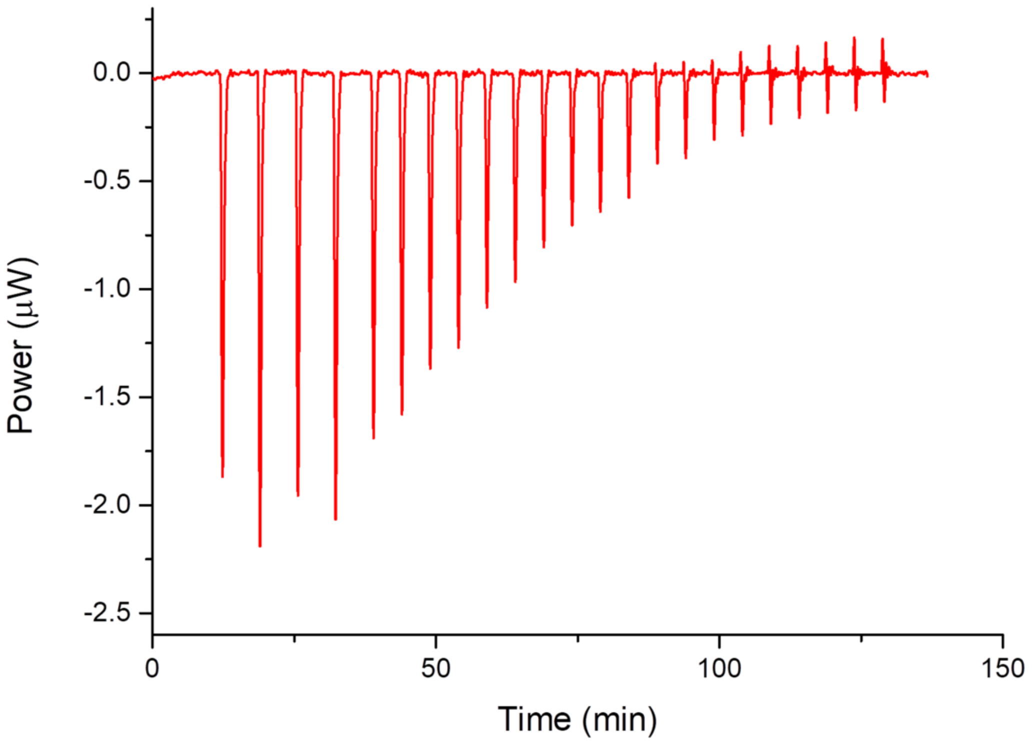


**Figure S7.** ITC titration of tetracycline 2 mM into MedeaC4dod 0.2 mM at 25 °C in neutral aqueous solution (pH 7.2, MOPS).

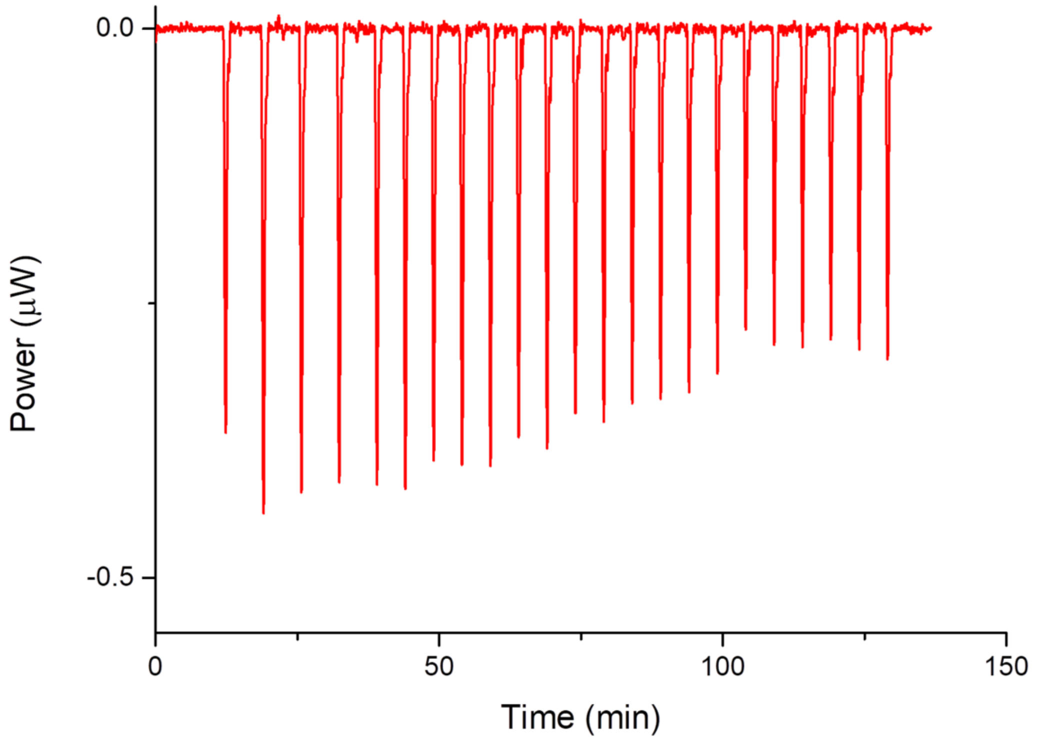


**Figure S8.** ITC titration of ofloxacin 2 mM into MedeaC4dod 0.2 mM at 25 °C in neutral aqueous solution (pH 7.2, MOPS).

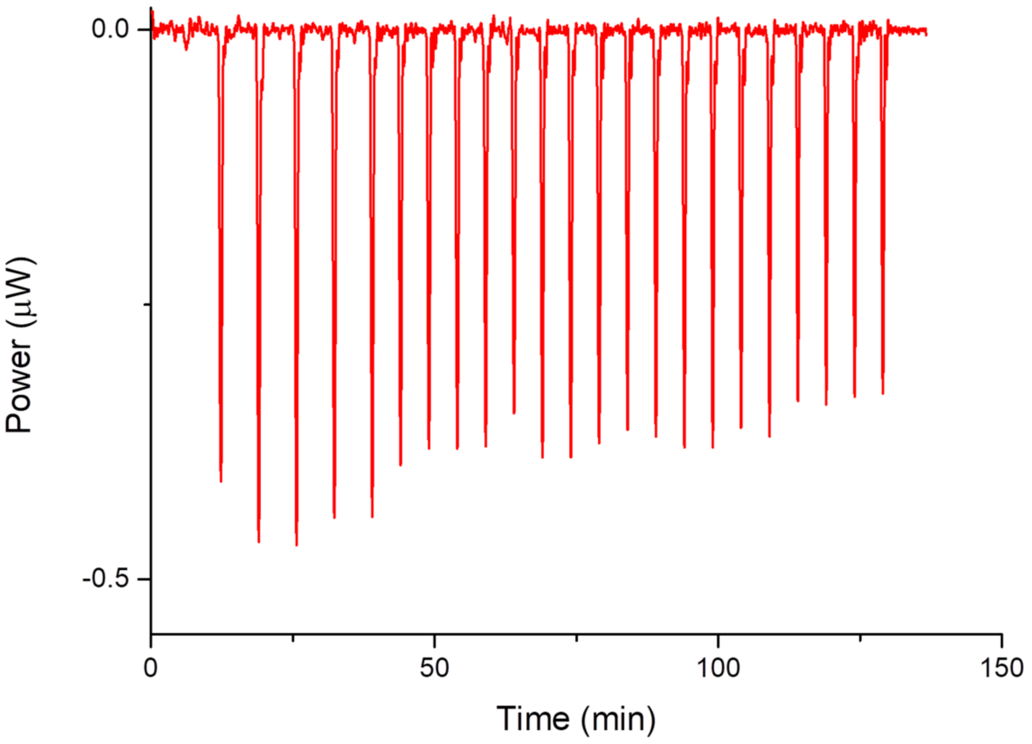


**Figure S9.** ITC titration of ofloxacin 2 mM into CholineC4dod 0.2 mM at 25 °C in neutral aqueous solution (pH 7.2, MOPS).

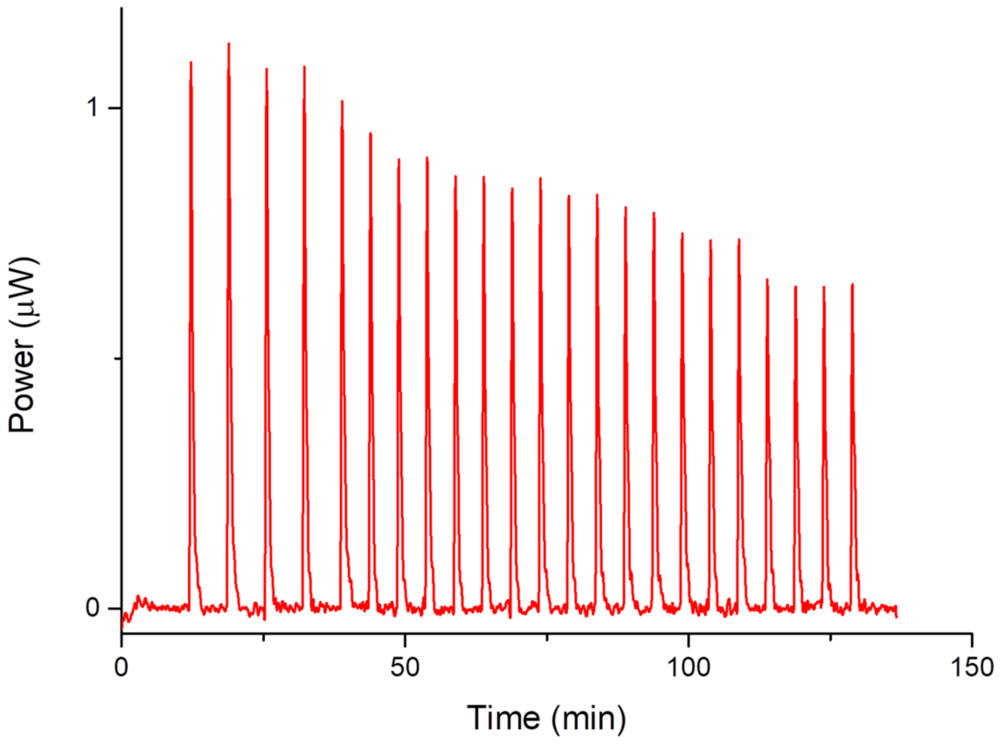


**Figure S10.** ITC titration of chloramphenicol 2 mM into MedeaC4dod 0.2 mM at 25 °C in neutral aqueous solution (pH 7.2, MOPS).

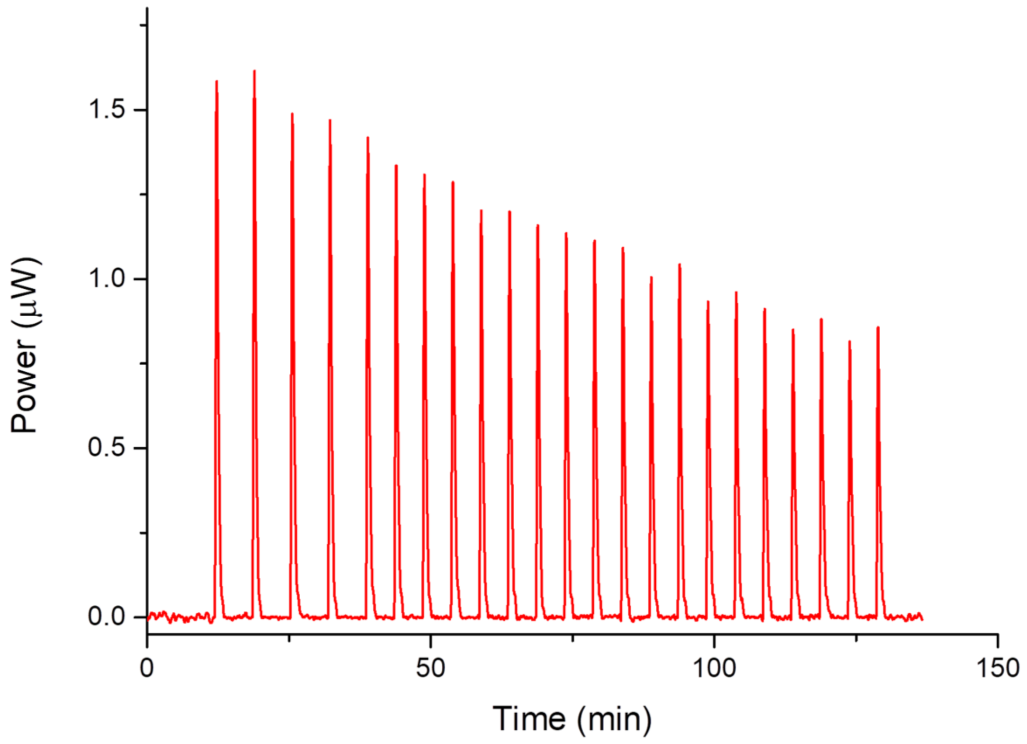


**Figure S11.** ITC titration of chloramphenicol 2 mM into CholineC4dod 0.2 mM at 25 °C in neutral aqueous solution (pH 7.2, MOPS).

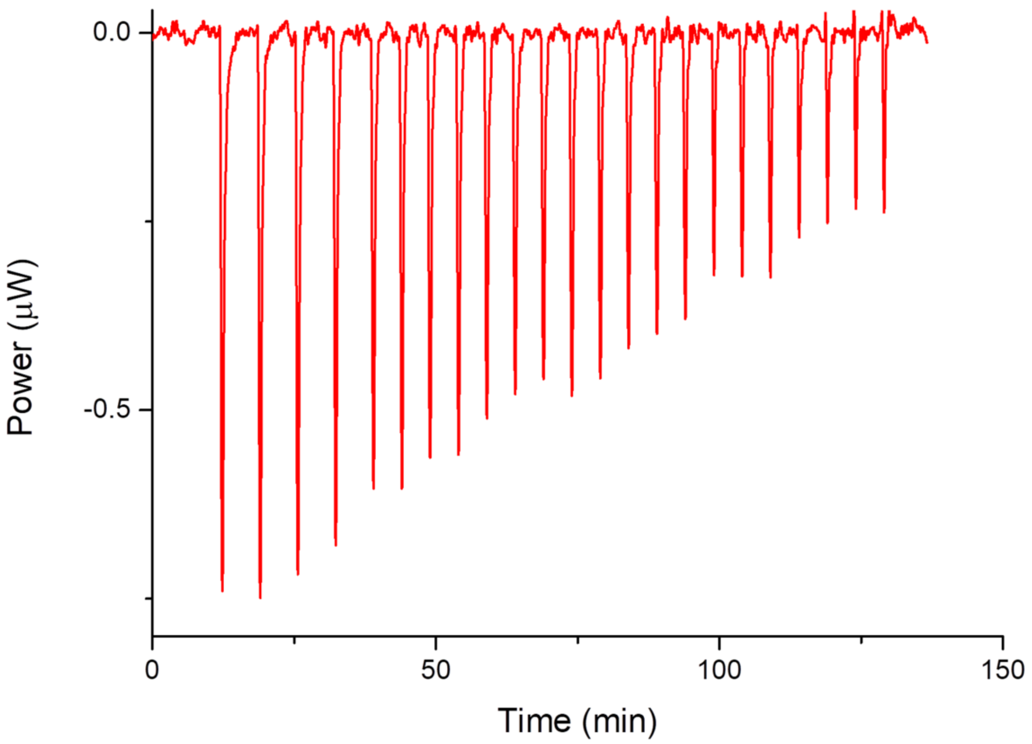


**Figure S12.** Typical "blank" experiment. Titration of tetracycline 2 mM into MOPS (pH 7.2)

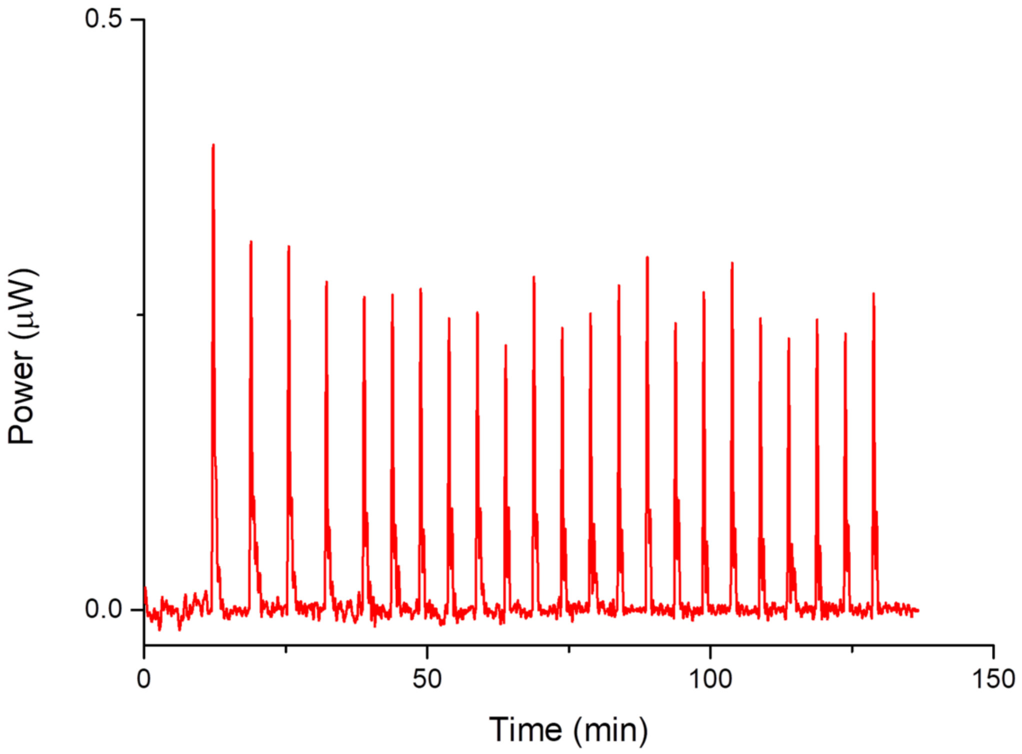


**Figure S13.** Typical "blank" experiment. Titration of ofloxacin 2 mM into MOPS (pH 7.2)

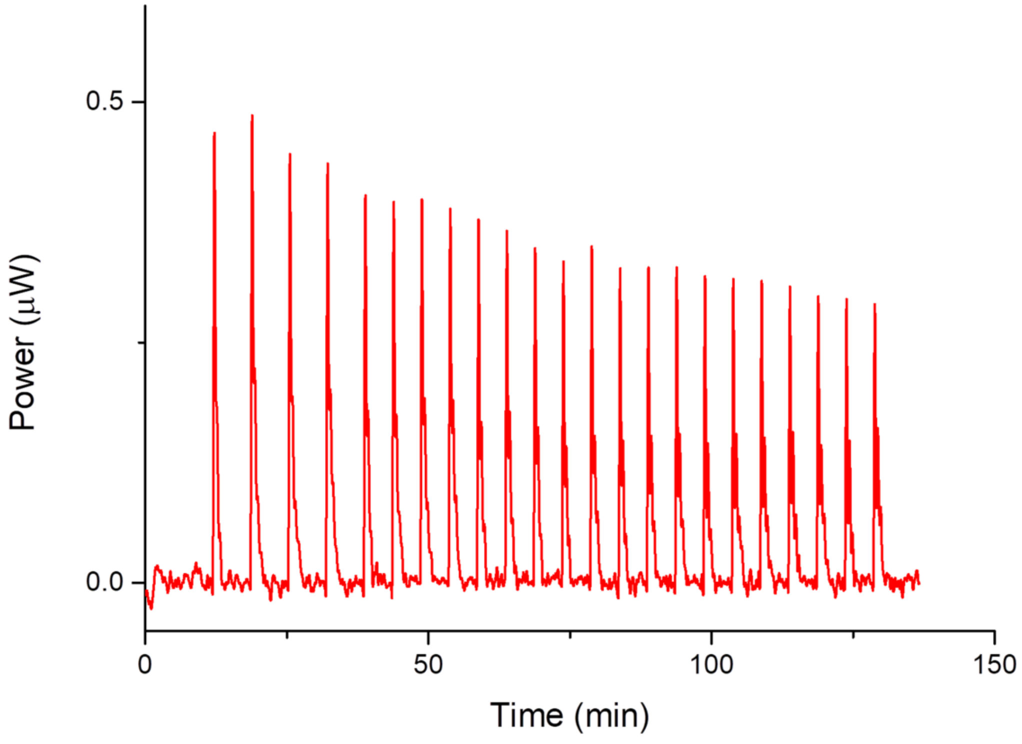


**Figure S14.** Typical "blank" experiment. Titration of chloramphenicol 2 mM into MOPS (pH 7.2)

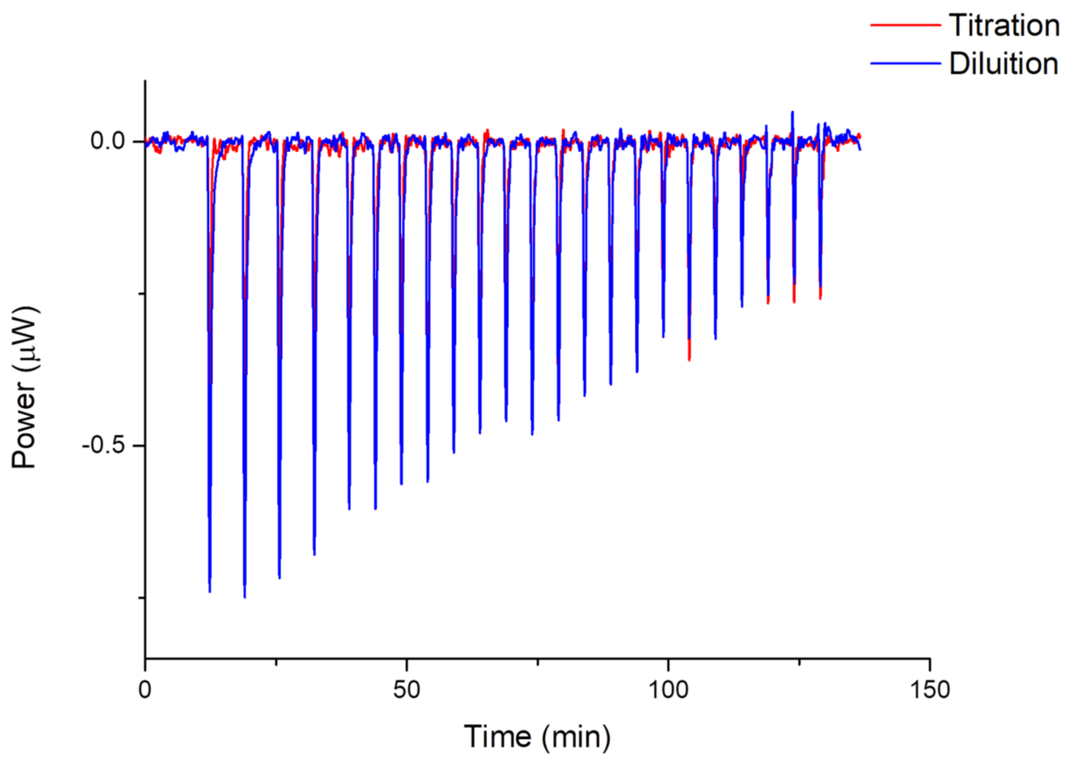


**Figure S15.** Overlap between host-guest titration and blank experiment (dilution) for the tetracycline (2 mM) / MedeaC4dod (2.5 μM) system.

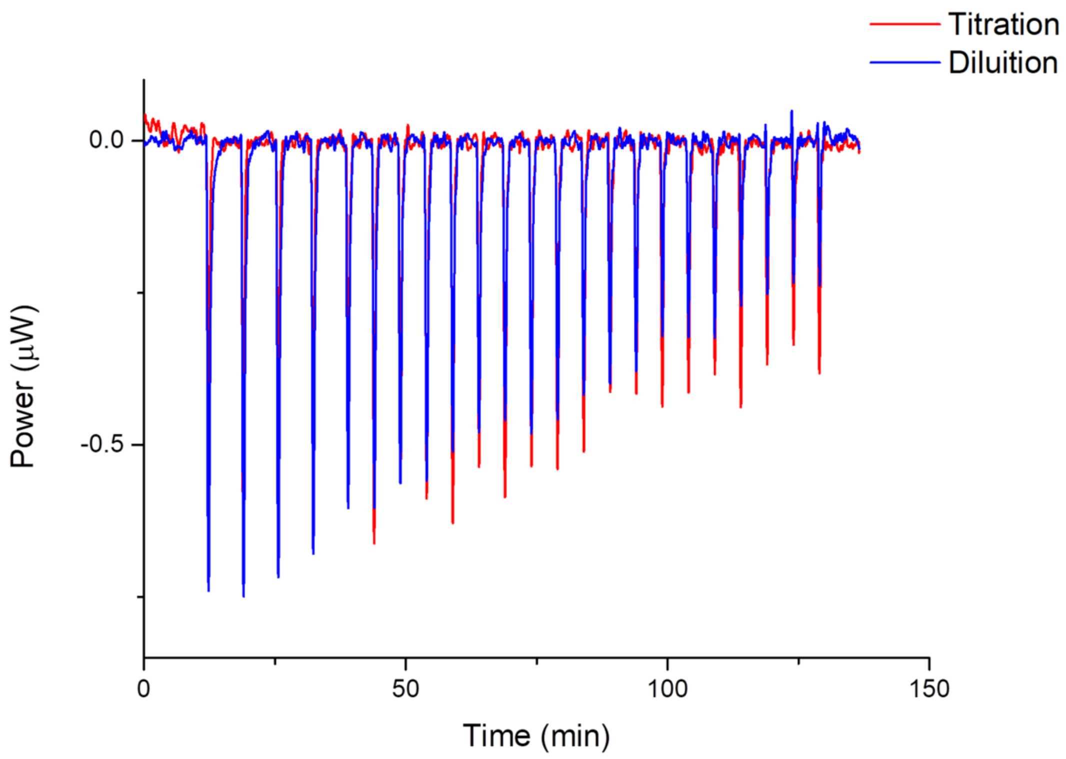


**Figure S16.** Overlap between host-guest titration and blank experiment (dilution) for the tetracycline (2 mM) / CholineC4dod (2.5 μM) system.

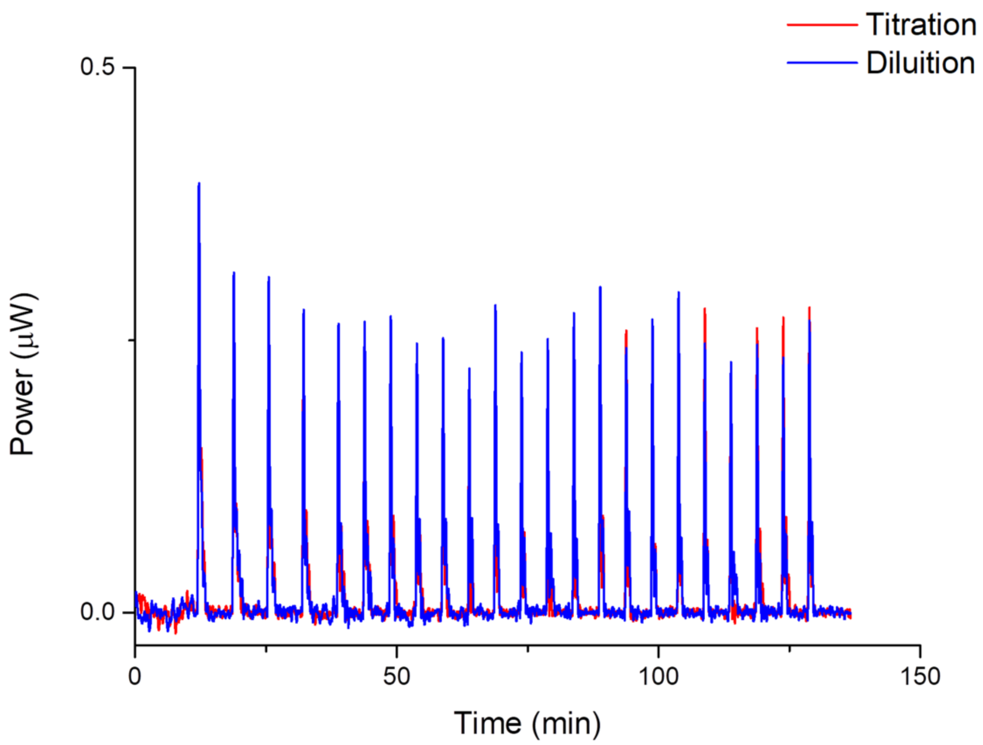


**Figure S17.** Overlap between host-guest titration and blank experiment (dilution) for the ofloxacin (2 mM) / MedeaC4dod (2.5 μM) system.

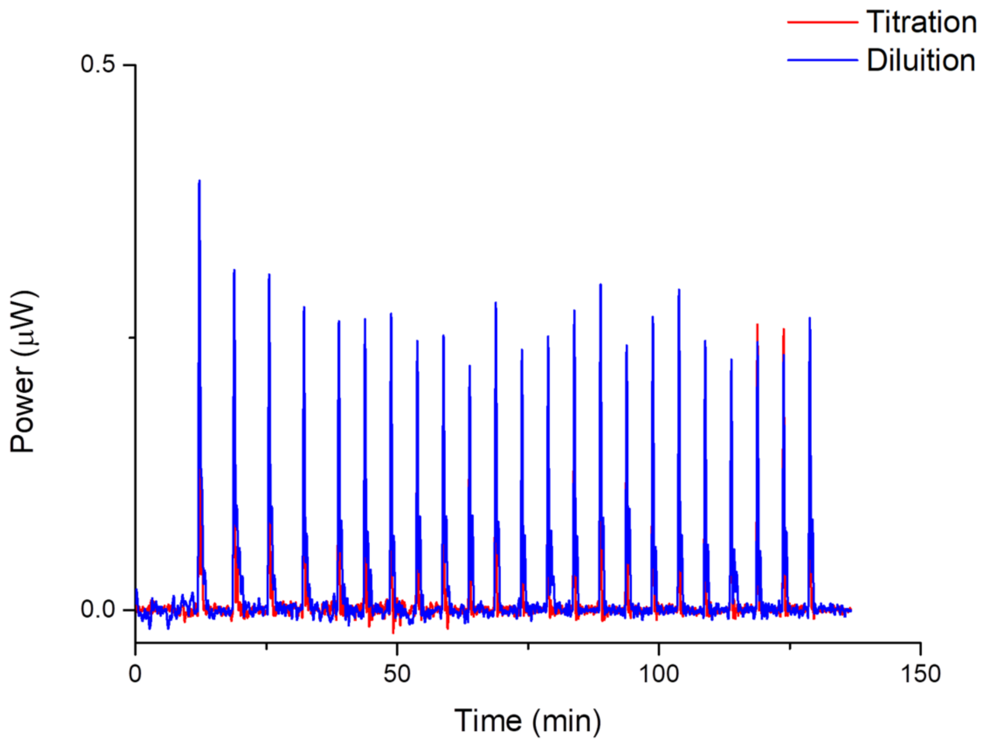


**Figure S18.** Overlap between host-guest titration and blank experiment (dilution) for the ofloxacin (2 mM) / CholineC4dod (2.5 μM) system.

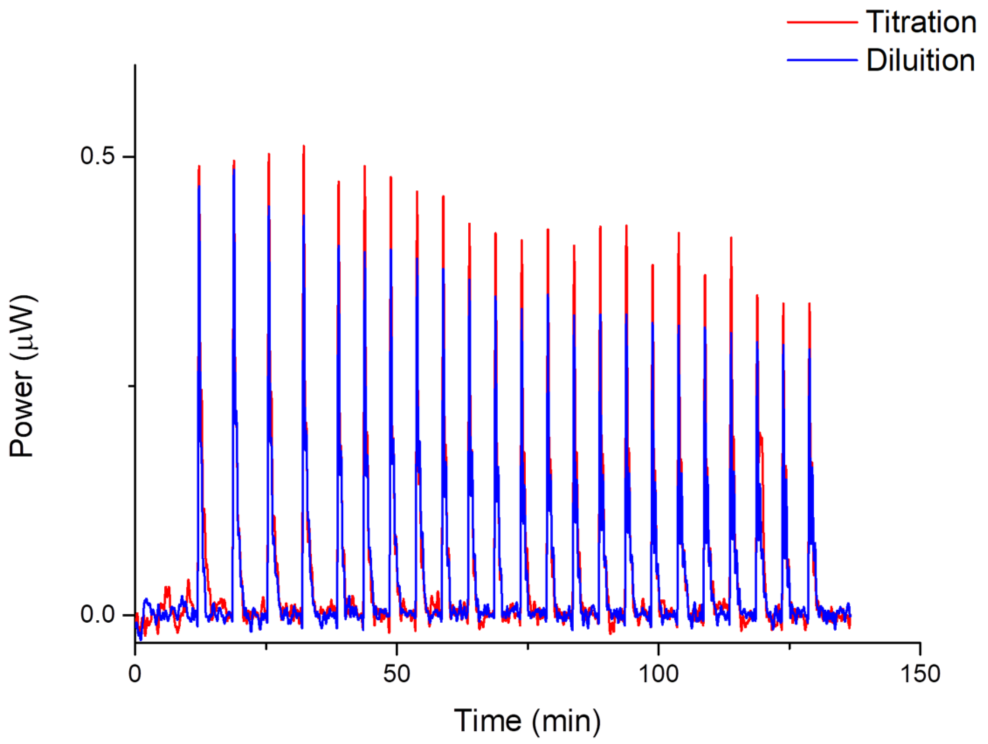


**Figure S19.** Overlap between host-guest titration and blank experiment (dilution) for the chloramphenicol (2 mM) / MedeaC4dod (2.5 μM) system.

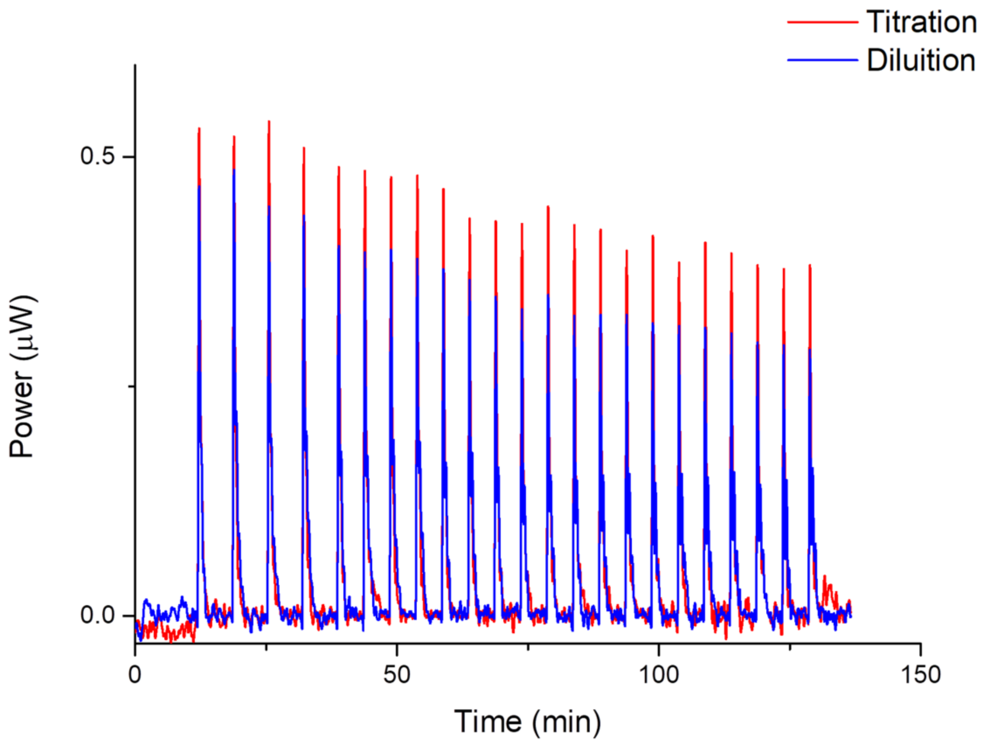


**Figure S20.** Overlap between host-guest titration and blank experiment (dilution) for the chloramphenicol (2 mM) / CholineC4dod (2.5 μM) system.

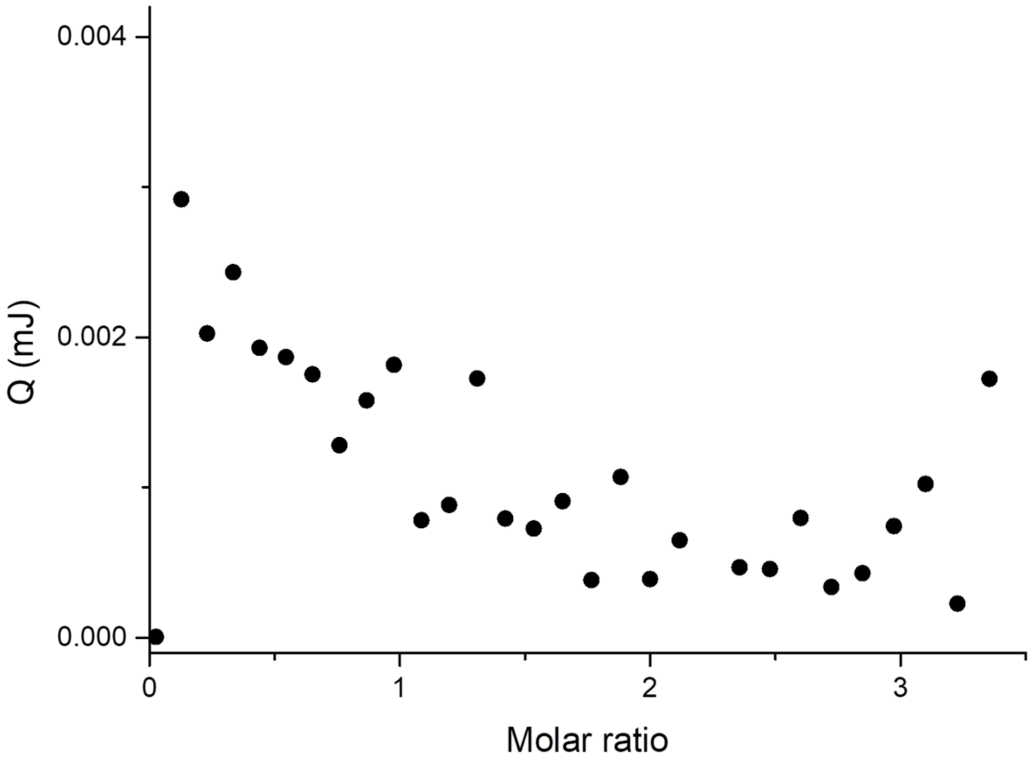


**Figure S21.** Net heat curve for the tetracycline (2mM) / MedeaC4prop (0.2 mM) system.

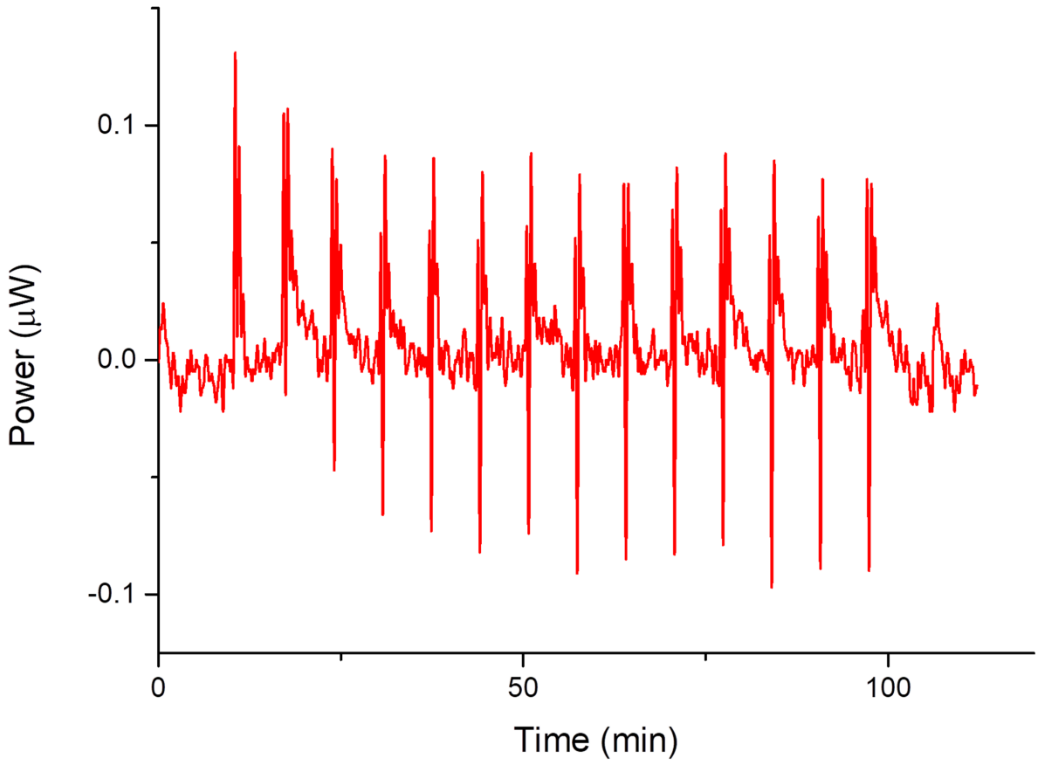


**Figure S22.** ITC titration of ofloxacin 2 mM into MedeaC4prop 0.2 mM at 25 °C in neutral aqueous solution (pH 7.2, MOPS).

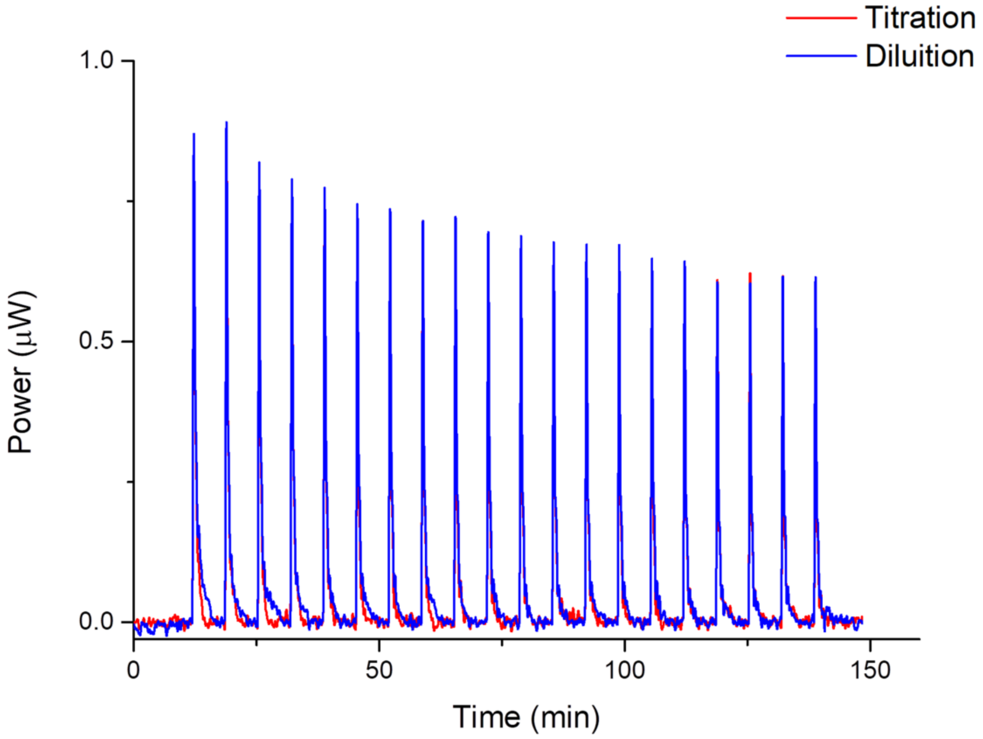


**Figure S23.** Overlap between host-guest titration and blank experiment (dilution) for the chloramphenicol (9 mM) / MedeaC4prop (0.5 mM) system.

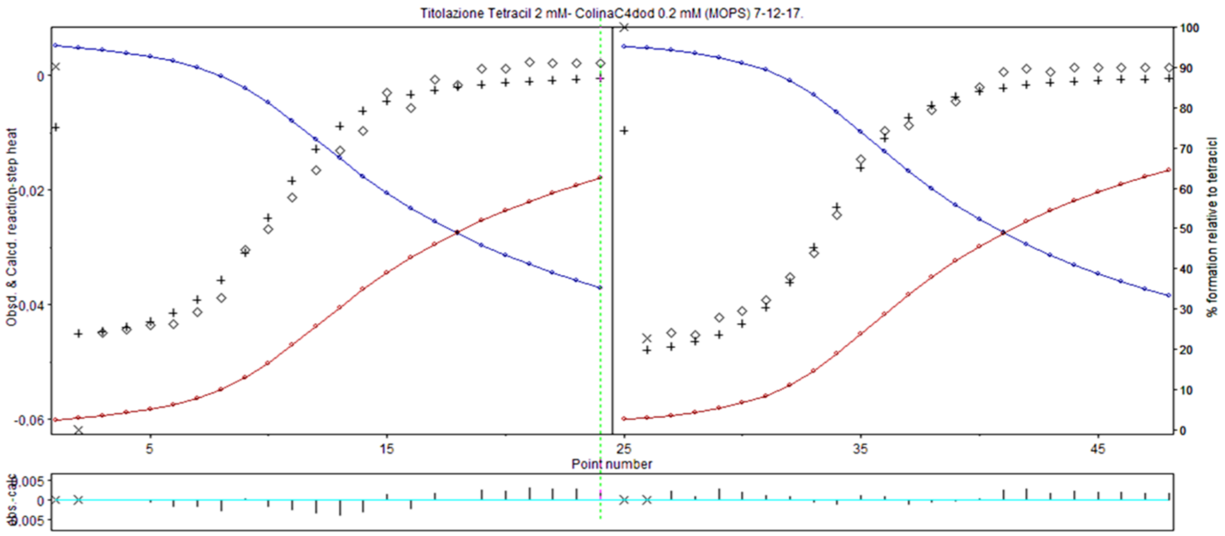


**Figure S24.** HypCal output for the complexation of tetracycline with CholineC4dod micellar aggregate. Experimental heats, diamonds; calculated heats, crosses. Residuals (observed - calculated values) are shown below the curves.

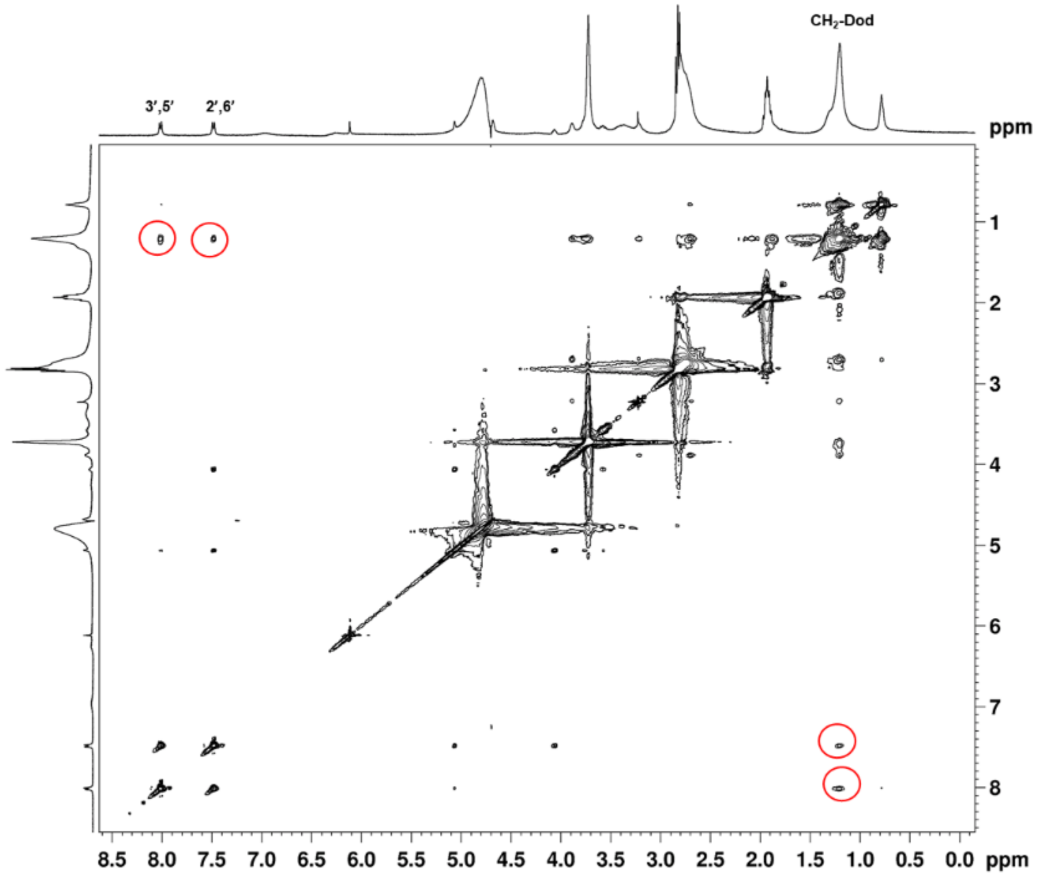


**Figure S25.** 2D-NOESY-NMR spectrum of the chloramphenicol (2.1 mM)/CholineC4dod (1.4 mM) system (MOPS buffer in $D_2O$, 297 K)

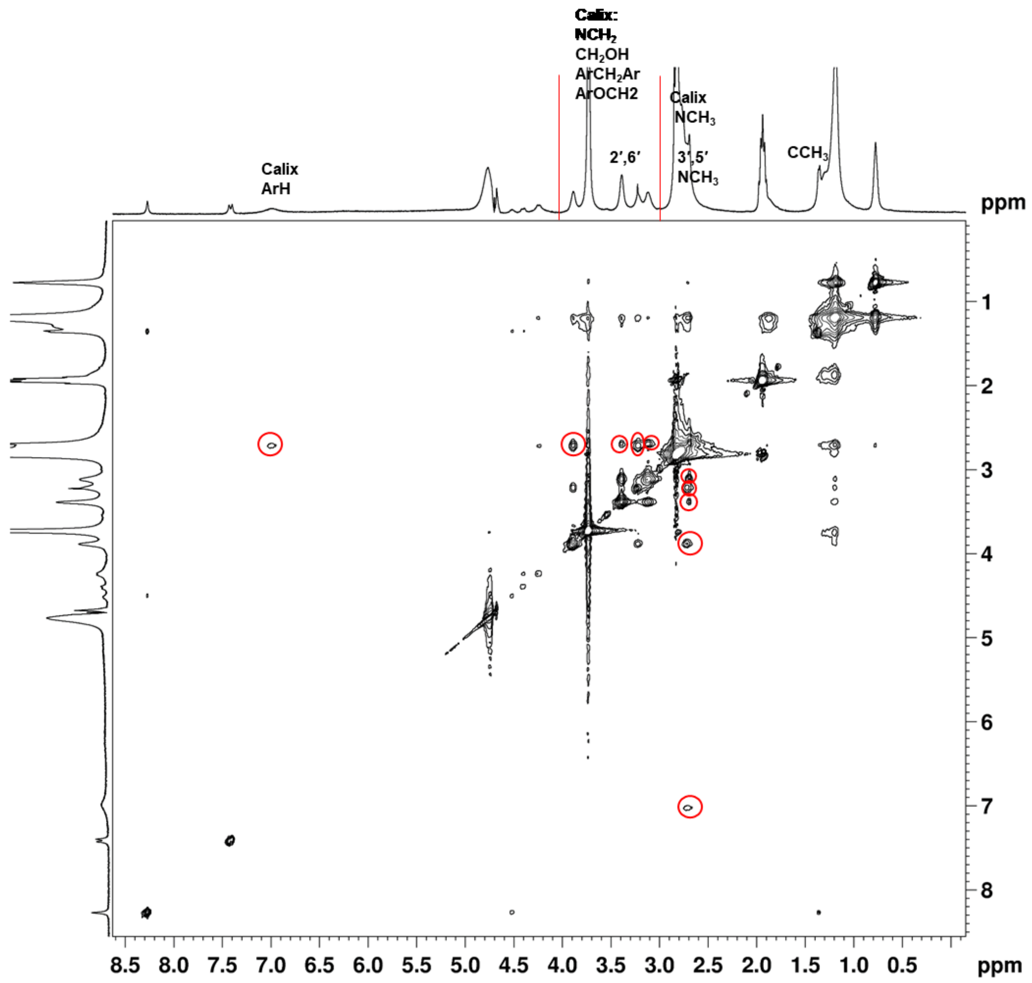


**Figure S26.** 2D-NOESY-NMR spectrum of the ofloxacin (2.1 mM)/CholineC4dod (1.4 mM) system (MOPS buffer in $D_2O$, 297 K)
